# The m^6^A reader YTHDC1 and the RNA helicase DDX5 control the production of rhabdomyosarcoma-enriched circRNAs

**DOI:** 10.1038/s41467-023-37578-7

**Published:** 2023-04-05

**Authors:** Dario Dattilo, Gaia Di Timoteo, Adriano Setti, Andrea Giuliani, Giovanna Peruzzi, Manuel Beltran Nebot, Alvaro Centrón-Broco, Davide Mariani, Chiara Mozzetta, Irene Bozzoni

**Affiliations:** 1 Department of Biology and Biotechnology Charles Darwin, Sapienza University of Rome, Rome, 00185 Italy; 2 Center for Life Nano- & Neuro-Science@Sapienza, Fondazione Istituto Italiano di Tecnologia (IIT), Rome, 00161 Italy; 3 Center for Human Technologies@Istituto Italiano di Tecnologia (IIT), Genoa, 16152 Italy; 4 Institute of Molecular Biology and Pathology (IBPM), National Research Council (CNR) of Italy, Rome, Italy

**Keywords:** Alternative splicing, RNA modification, Long non-coding RNAs, Paediatric cancer

## Abstract

N6-Methyladenosine (m^6^A) is well-known for controlling different processes of linear RNA metabolism. Conversely, its role in the biogenesis and function of circular RNAs (circRNAs) is still poorly understood. Here, we characterize circRNA expression in the pathological context of rhabdomyosarcoma (RMS), observing a global increase when compared to wild-type myoblasts. For a set of circRNAs, such an increase is due to the raised expression of the m^6^A machinery, which we also find to control the proliferation activity of RMS cells. Furthermore, we identify the RNA helicase DDX5 as a mediator of the back-splicing reaction and as a co-factor of the m^6^A regulatory network. DDX5 and the m^6^A reader YTHDC1 are shown to interact and to promote the production of a common subset of circRNAs in RMS. In line with the observation that YTHDC1/DDX5 depletion reduces RMS proliferation, our results provide proteins and RNA candidates for the study of rhabdomyosarcoma tumorigenicity.

## Abstract (web summary)

Rhabdomyosarcoma (RMS) is the most diffused soft tissue sarcoma in children and adolescents. Herein, the authors identify the m^6^A machinery and the RNA helicase DDX5 as factors responsible for the increase of a subset of circRNAs in RMS, providing protein and RNA candidates for the study of its tumorigenicity.

## Introduction

The complex family of non-coding RNAs includes many classes of molecules with different sizes, functions, and processing mechanisms. Among the species recently entered into this family, circular RNAs (circRNAs) represent an interesting class not only for the multitude of functions they play but also because they originate through a form of alternative splicing named “back-splicing”. The peculiarity of this reaction is that a downstream 5’ donor splice site is joined to an upstream 3’ acceptor splice site, leading to the circularization of the intervening exon(s) and the generation of a back-splicing junction (BSJ), which is unique to the circular form^1–3^. Even though circRNA production is a feature shared by many genes, the regulation of their biogenesis is still poorly understood. The few studies on the topic indicate that the back-splicing reaction requires the formation of a loop between introns flanking the circularizing exon(s) and this can be mediated either *in cis* by base pairing between inverted sequences present within such introns^4–6^, or in trans by the recruitment of RNA binding proteins (RBPs) which favor the juxtaposition of circRNA splice sites. To date, some RBPs regulating circRNA biogenesis have been identified, including the splicing factors QKI^7^, MBL^8^, FUS^9^, NOVA2^10^, and multiple hnRNPs and SR proteins^11–13^.

Recently, also RNA modifications have been discovered to participate in circRNA production, particularly N6-methyladenosine (m^6^A). m^6^A is the most abundant internal messenger RNA (mRNA) modification^14^, mainly deposited by the writer METTL3^15–17^ which acts as part of a larger complex called m^6^A-METTL-associated complex (MACOM). Also, circRNAs can be decorated with m^6^A and exhibit methylation patterns that are different from the ones of their linear mRNA counterparts^18^, suggesting that this mark might play a role in differentiating the production or function of a circular *versus* a linear transcript. Indeed, a recent study showed that m^6^A can specifically promote circRNA back-splicing^1^ through the recruitment of the nuclear reader YTHDC1^19^, which drives the precursor transcript towards the maturation in a circular molecule.

In this work, we addressed circRNA expression in the context of rhabdomyosarcoma (RMS), the most common type of soft tissue sarcoma in children and adolescents^20,21^, arising from skeletal myoblast-like cells unable to complete the differentiation program^22,23^. RMS is classified into two major subtypes: embryonal RMS (ERMS) is the most diffused form, generally associated with a favorable outcome, while alveolar RMS (ARMS) is more aggressive and tends to metastasize^24^.

Recent studies identified an oncogenic role for specific circRNAs in RMS^25–27^. Particularly, circZNF609 was shown to promote RMS progression via the regulation of microtubule dynamics. Interestingly, circZNF609 was shown to require m^6^A modifications for its efficient biogenesis, and, notably, its levels increased in RMS paralleling the upregulation of the m^6^A reader YTDHC1^19^.

In this study, we demonstrated that RMS lines have increased levels of the m^6^A machinery and that this correlates with the upregulation of a large fraction of circRNAs, and in particular of a specific group in which the linear counterparts do not vary in the same direction. We observed that m^6^A-dependent regulation is not only important for sustaining circRNA expression in RMS, but also participates in tumor progression, with a possible contribution of m^6^A-modified circRNA species. Finally, we found that the reader YTDHC1 and the helicase DDX5 promote the back-splicing reaction of a set of m^6^A-containing circRNAs, suggesting that the modulation exerted by their activity may act as an oncogenic feature in RMS.

## Results

### CircRNA levels increase in RMS

Despite RMS being a well-studied tumor, the profile of circRNAs expressed in this system has not been characterized so far. Therefore, we performed total RNA-seq on samples from RD or RH4 cell lines, representative of the ERMS or ARMS subtypes, respectively. As a healthy control, we also sequenced RNA from wild-type human myoblasts.

By applying CIRI2 algorithm^28^ we identified 2897 circRNAs expressed in wild-type myoblasts, 4935 in RD cells, and 3811 in RH4 cells (Supplementary Fig. 1a). Interestingly, unlike linear mRNAs which were largely shared among the three lines (70%), only 19% of the detected circRNAs were common to all systems. This observation indicated that circRNA expression in our systems tends to be much more cell line specific than the one of linear RNAs.

**Fig. 1:**
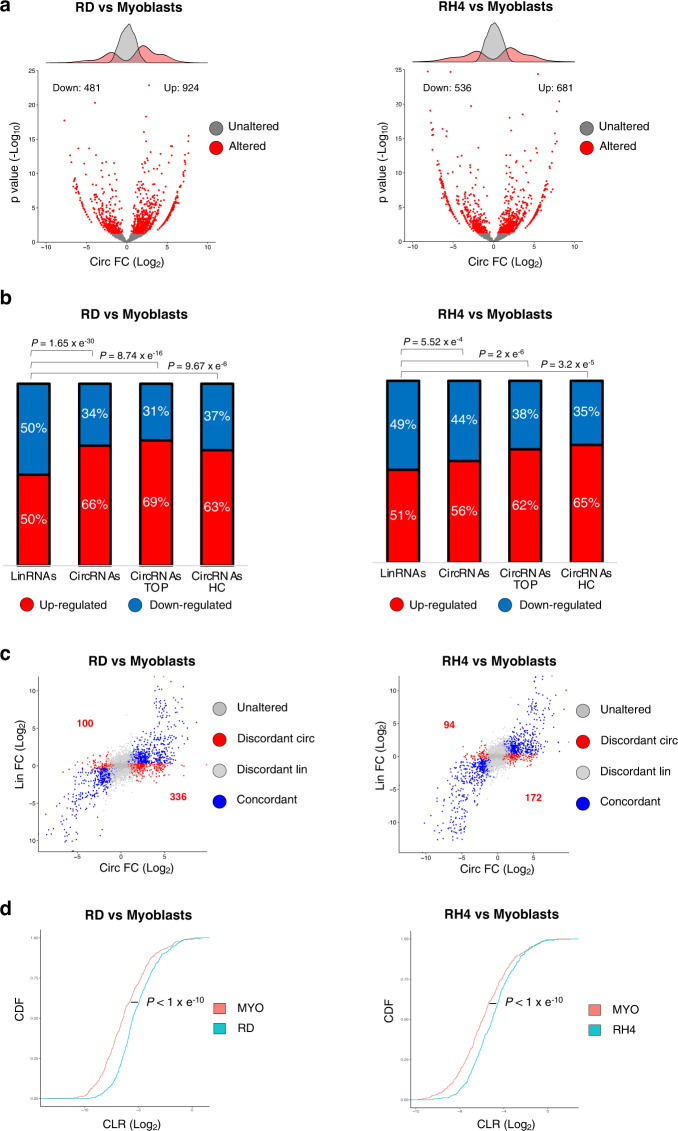
CircRNA levels increase in RMS. **a** Volcano plots showing, for each circRNA identified in the RNA-Seq experiment, the log_2_ fold change and the −log_10_ *p* value either in the comparison between RD (left panel) or RH4 (right panel) cell lines and wild-type myoblasts. The distributions of the fold change values are shown above the volcano plots. Significantly altered circRNAs (*p* value < 0.05) or unaltered circRNAs are indicated by red or gray dots, respectively. **b** Stacked bar charts with percentage of down- and upregulated linear (“LinRNAs”), circRNAs (“CircRNAs”), top 20% expressed circRNAs (“CircRNAs TOP”) or “*high-confidence*” circRNAs (“CircRNAs HC”) in the comparison between RD and wild-type myoblasts (left panel) or between RH4 and wild-type myoblasts (right panel). *P* values for the differences between proportions were calculated using Fisher exact two-tailed test. **c** Scatter plots showing, for each circRNA identified in the RNA-seq experiment, the log_2_ fold change along with that of its cognate linear RNA, in the comparison between RD (left panel) or RH4 (right panel) cell lines and wild-type myoblasts. Significantly deregulated circRNAs are indicated by blue dots when their linear counterpart is deregulated in the same direction (“Concordant”) and by red dots when the linear is either unaltered or deregulated in the opposite direction (“Discordant circ”); significantly deregulated linRNAs are indicated by light gray dots when their circular counterpart is unaltered (“Discordant lin”); unaffected circRNAs are indicated by dark gray dots when their linear counterpart is not altered (“Unaltered”). **d** Cumulative distribution function of the circular *versus* the linear isoform expression (“CLR”) in myoblasts (red) and RD (blue, left panel) or RH4 (blue, right panel). *P* values for the differences between cumulative distribution functions were calculated using Kolmogorov–Smirnov two-tailed test. Source data are provided as a Source Data file.

Differential expression analysis comparing each RMS line with the wild-type myoblasts identified 924 and 681 circRNAs upregulated in RD and RH4, respectively. Instead, the downregulated circRNAs were 481 and 536 (Fig. 1a, Supplementary Data 1). Interestingly, comparing RMS and control myoblasts, while the amount of up- and downregulated linear mRNAs was the same, circRNAs displayed an evident predominance of upregulated species in cancer cells (Fig. 1b, Supplementary Fig. 1b).

Given that circRNAs are usually expressed at low levels, in order to exclude artifacts due to circRNAs barely detected in one of the two groups, we refined our analysis by addressing the most expressed species (top 20%). We found that, with respect to the total, this fraction displayed an even higher percentage of upregulated circRNAs in RMS *versus* the control myoblast cell line, reaching the value of 68.83% in RD and 62.19% in RH4 (Fig. 1b, Supplementary Fig. 1c).

Moreover, since circRNA detection tools can have high false positive rates, we queried two additional algorithms (circExplorer2^29^ and DCC^30^) and we defined as “*high-confidence*” the species identified by all the tools. Even if such group only represents a fraction of the initial dataset (15.5–17% with respect to the CIRI2 algorithm, mostly due to circExplorer2 which considers only annotated splice junctions (Supplementary Fig. 1e), it was selected as the most stringent and reliable sample. Interestingly, the increased number of circRNAs observed in RMS cell lines was again more evident when looking at this group (63.44% in RD and of 64.76% in RH4 (Fig. 1b, Supplementary Fig. 1d), further supporting previous observations.

Since linear RNA species are much more abundant than circRNAs, we ruled out the presence of a bias in comparing two groups of different sizes (the circOme and the linear transcriptome) by restricting the analysis to genes producing both linear and circular transcripts. Among the deregulated species, RNAs were defined as “*concordant*” if circular and linear isoforms varied in the same direction, while all the other cases were termed as “*discordant*”. When analyzing “*discordant*” species—for which transcriptional control is likely to be excluded as the source of different isoform production—we observed a visible enrichment of upregulated circRNAs in RMS which was not mirrored by linear RNAs (Fig. 1c, Supplementary Fig. 1b). The specific increase in circRNA levels was also observed when analyzing the “*concordant”* species, where both the circular and the linear counterparts increased in RMS. To evaluate the relative abundance of the two isoforms, we calculated the circular-to-linear *ratio* (“CLR”). Analysis of the cumulative distribution function (CDF) showed higher CLR in RMS cells (Fig. 1d).

Altogether, these results indicate that RMS cell lines display enhanced circularization of a consistent number of circRNAs, suggesting that tumor cells express factors that are able to specifically favor circRNA levels over their linear counterparts.

### m^6^A factors are altered in RMS and sustain its proliferation and migration rate

**Fig. 2:**
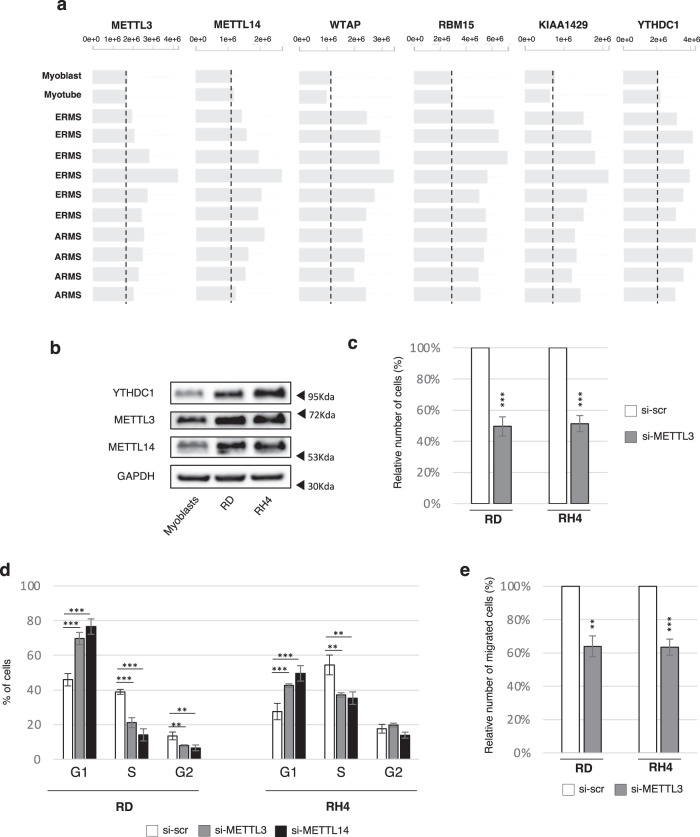
m^6^A factors are altered in RMS and sustain its proliferation and migration rate. **a** Protein levels for several components of the MACOM complex, as well as for the reader YTHDC1, in orthotopic RMS patient-derived xenografts, compared to normal myoblasts and myotubes. Data derive from https://pecan.stjude.cloud/proteinpaint/study/RHB2018. A vertical dashed line indicates the level of protein expression of each gene in normal myoblasts. **b** Representative western blot to evaluate the levels of METTL3, METTL14, and YTHDC1 in wild-type myoblasts, RD and RH4 cell lines; GAPDH was used as loading control. *n* = 3 biologically independent replicates. **c** Relative number of cells upon control treatment (“si-scr”) or METTL3 depletion (“si-METTL3”) in RD or RH4 cell lines 48 hrs post transfection. Data are represented as mean percentage of cells ± SD. *n* = 4 biologically independent replicates. **d** Cell cycle analysis by FACS of RD or RH4 cells either upon control treatment (“si-scr”), METTL3 knock-down (“si- METTL3”), or METTL14 knock-down (“si- METTL14”). Data are represented as mean percentage of cells in each cell cycle phase ± SD. *n* = 4 biologically independent replicates. **e** Relative number of cells/field measured after transwell-migration assay with DAPI staining upon control treatment (“si-scr”) or METTL3 depletion (“si-METTL3”) in RD and RH4 cells. Data are represented as mean percentage of migrated cells ± SEM. *n* = 4 biologically independent replicates. Where statistical analysis was performed, the *ratio* of each sample *versus* its experimental control was tested by a two-tailed unpaired Student’s *t* test (**c**, **e**) with correction for multiple test comparison (FDR Benjamini–Hochberg) (**d**). * indicates a test-derived *p* value < 0.05, ** indicate a *p* value < 0.01, and *** a *p* value < 0.001. Source data are provided as a Source Data file.

We previously showed that the biogenesis of a specific set of circRNAs is enhanced by m^6^A modifications which in turn require recognition by the reader YTHDC1^19^. Since circularization is augmented in RMS, we hypothesized that such a process might be due to the deregulation of m^6^A factors. We first consulted the Integrated Rhabdomyosarcoma database of the St. Jude Children’s Research Hospital, which collects proteomic data from orthotopic RMS patient-derived xenografts (O-PDX), as well as from human myoblasts and myotubes. As shown in Fig. 2a, the levels of the MACOM complex components (METTL3, METTL14, WTAP, RBM15, and KIAA1429) and of the reader YTHDC1 are higher in RMS patients when compared to healthy controls. Coherently, in RMS cell lines we validated the upregulation of METTL3, METTL14, and YTHDC1 both at the protein and RNA levels (Fig. 2b, Supplementary Fig. 2a). As a consequence, RMS cell lines might have higher levels of m^6^A-modified RNA as well as an enhanced binding of YTHDC1, suggesting that they may promote the formation of circRNA species in the tumor.

m^6^A has been shown to participate in the regulation of multiple processes, including cell proliferation. Since the m^6^A writer METTL3 is upregulated in RMS, we speculated that it could play a role in tumor growth. To test this hypothesis, we depleted METTL3 in RD and RH4 cell lines with a siRNA-based approach (Supplementary Fig. 2b) and observed an ~50% reduction in the total number of cells after 48 hrs transfection (Fig. 2c, Supplementary Fig. 2c). In order to evaluate the global decrease in m^6^A levels upon METTL3 depletion, we performed m^6^A CLIP, observing that the recovery of a known m^6^A-containing circRNA (circZNF609^19^) was reduced in such condition (Supplementary Fig. 2d).

To see whether the strong decrease in cell number was due to an alteration of the cycle progression, we conducted FACS analysis with propidium iodide (PI) staining, allowing us to discriminate fractions of cells in each cell cycle phase according to their DNA content. As shown in Fig. 2d, both RMS cell lines depleted for METTL3 experienced a strong increase in the G1 phase with a corresponding decrease of the S phase. Moreover, the RD cell line also exhibited a downregulation of the G2 phase upon METTL3 depletion, indicating that this factor might regulate cell cycle progression at multiple stages.

Such phenotype was mirrored when depleting METTL14, another core component of the MACOM complex (Fig. 2d, Supplementary Fig. 2e), indicating that cell cycle regulation mediated by METTL3 is likely due to its methyltransferase activity. In accordance with previous studies reporting that METTL3 and METTL14 stabilize each other in a heterodimer complex^31^, the levels of both factors were reduced upon their reciprocal depletion (Supplementary Fig. 2e). Finally, transwell-migration assays revealed that METTL3 depletion also reduced the migration ability of RMS cells, pointing out a role of m^6^A-dependent pathways in sustaining different aspects of RMS tumorigenicity (Fig. 2e, Supplementary Fig. 2f).

### YTHDC1 depletion downregulates circRNAs in RMS

Since YTHDC1 was previously shown to positively regulate the biogenesis of a specific set of circRNAs^19^, in order to test whether the higher expression of circRNAs in RMS could be attributed to the increased levels of this protein, we performed total RNA-seq of RD and RH4 cell lines upon YTHDC1 knock-down (Supplementary Fig. 3a).

**Fig. 3:**
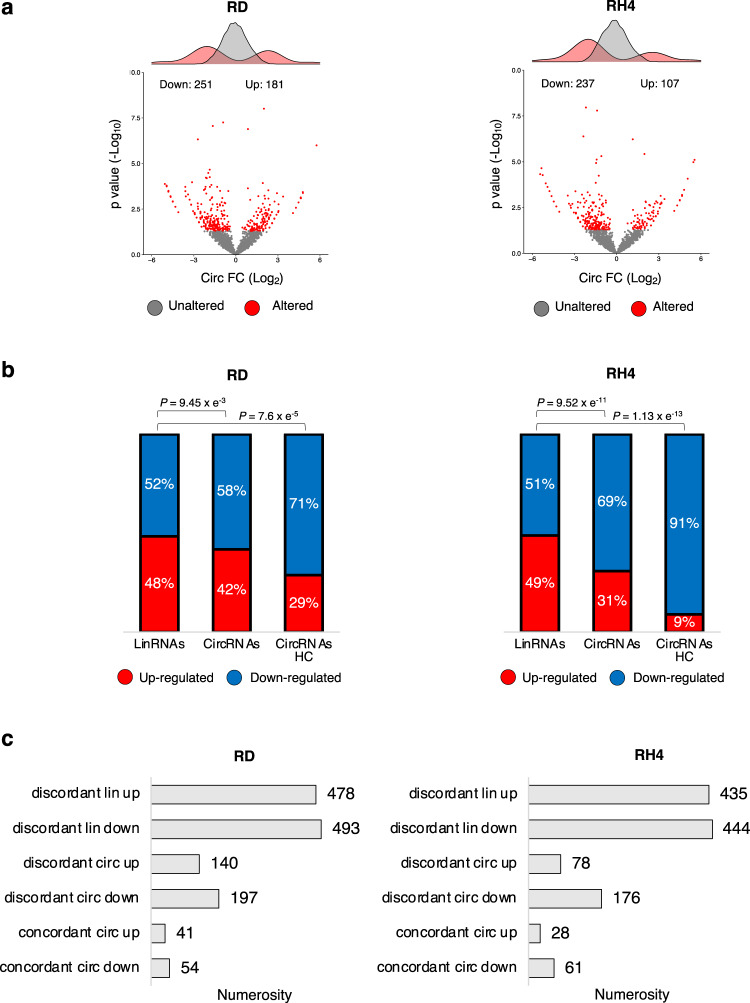
YTHDC1 depletion downregulates circRNAs in RMS. **a** Volcano plots showing for each circRNA identified in the RNA-seq experiment the log_2_ fold change and the −log_10_ *p* value upon YTHDC1 knock-down in RD (left panel) or in RH4 (right panel) cells. The distributions of the fold change values are shown above the volcano plots. Significantly altered circRNAs (*p* value < 0.05) or unaltered circRNAs are indicated by red or gray dots, respectively. See the methods section for statistical analyses details. **b** Stacked bar charts with percentage of down- and upregulated linear (“LinRNAs”), circRNAs (“CircRNAs”), or “high-confidence” circRNAs (“CircRNAs HC”) upon YTHDC1 knock-down in RD (left panel) or RH4 (right panel) cell lines. *P* values for the differences between proportions were calculated using Fisher exact two-tailed test. **c** Bar-plots depicting the numerosity of different scenarios of deregulation in RD (left panel) or in RH4 (right panel) cells upon YTHDC1 knock-down. Source data are provided as a Source Data file.

Differential expression analysis identified 251 downregulated circRNAs in RD and 237 in RH4, *versus* 181 upregulated in RD and 107 in RH4 (Fig. 3a, Supplementary Data 1). Notably, while the deregulated circRNAs predominantly belonged to the category of the downregulated species, the linear mRNAs were equally distributed between up- and downregulated (Fig. 3b).

Further supporting the specificity of YTHDC1 activity on the production of circRNAs, we observed that downregulated circRNAs mainly belonged to the “*discordant”* class in both RMS cell lines (Fig. 3c, Supplementary Fig. 3b), especially when considering only “*high-confidence*” circRNAs (Fig. 3b, Supplementary Fig. 3c, d).

Altogether, these data suggest that YTHDC1-mediated regulation of circRNA biogenesis in RMS is largely independent of changes in the transcription levels of the host gene.

### DDX5 helicase controls circRNA expression and interacts with YTHDC1

**Fig. 4:**
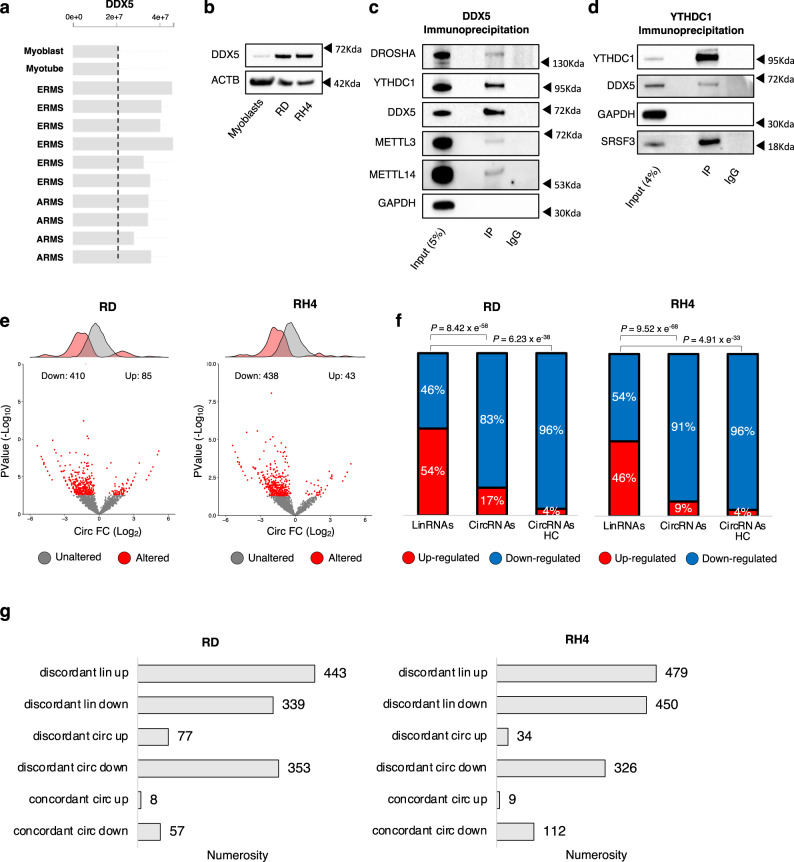
DDX5 helicase controls circRNA expression and interacts with YTHDC1. **a** Protein levels for DDX5 in orthotopic RMS patient-derived xenografts, as compared to normal myoblasts and myotubes. Data derive from https://pecan.stjude.cloud/proteinpaint/study/RHB2018. A vertical dashed line indicates the level of protein expression of DDX5 in normal myoblasts. **b** Representative western blot to evaluate the levels of DDX5 in wild-type myoblasts, RD, and RH4 cell lines; ACTB was used as loading control. *n* = 3 biologically independent replicates. **c** Representative western blot analysis of DDX5 immunoprecipitation from whole-cell lysate in RD cells. The percentage of input is indicated. DROSHA and GAPDH were used as a positive and negative control for the co-immunoprecipitation, respectively. *n* = 2 biologically independent replicates. **d** Representative western blot analysis of YTHDC1 immunoprecipitation from whole-cell lysate in RD cells. The percentage of input is indicated. SRSF3 and GAPDH were used as a positive and negative control for the co-immunoprecipitation, respectively. *n* = 2 biologically independent replicates. **e** Volcano plots showing for each circRNA identified in the RNA-seq experiment the log_2_ fold change and the −log_10_ *p* value upon DDX5 knock-down in RD (left panel) or in RH4 (right panel) cells. The distributions of the fold change values are shown above the volcano plots. Significantly altered circRNAs (*p* value < 0.05) or unaltered circRNAs are indicated by red or gray dots, respectively. See the methods section for statistical analyses details. **f** Stacked bar charts with percentage of down- and upregulated linear (“LinRNAs”), circRNAs (“CircRNAs”), or “*high-confidence*” circRNAs (“CircRNAs HC”) upon DDX5 knock-down in RD (left panel) or RH4 (right panel) cell lines. *P*-values for the differences between proportions were calculated using Fisher exact two-tailed test. **g** Bar-plots depicting the numerosity of different scenarios of deregulation in RD (left panel) or in RH4 (right panel) cells upon DDX5 knock-down. Source data are provided as a Source Data file.

The observation that YTHDC1 sustains the levels of a set of circRNAs in RMS prompted us to look for other factors contributing to this regulation. It has been previously shown that back-splicing is dependent on the formation of RNA structures in the flanking introns of circularizing exons or on the binding of specific factors that favor the juxtaposition of the engaged splice junctions^2^. These processes might be regulated by proteins involved in alternative splicing or in the regulation of RNA conformation, such as helicases. The ATP-dependent RNA helicase DEAD box helicase 5 (DDX5) was previously reported not only to participate in many aspects of RNA processing^32,33^, but also to be involved in the regulation of myogenesis and RMS progression^34,35^. Moreover, recent studies have identified DDX5 in complex with METTL3^36,37^, suggesting a possible crosstalk with the m^6^A machinery. Interestingly, when comparing RMS O-PDXs samples to wild-type myoblasts we observed the increase of DDX5 protein levels (Fig. 4a). We validated such upregulation in RD and RH4 cell lines both at the protein and RNA levels (Fig. 4b, Supplementary Fig. 4a).

DDX5 immunoprecipitation in RD cells revealed the presence of YTHDC1 in the IP fraction, as well as of the positive controls METTL3 and Drosha^38^ (Fig. 4c). The complementary immunoprecipitation of YTHDC1 further validated its interaction with DDX5 (Fig. 4d). In this case, SRSF3^39^ and GAPDH were used as positive and negative controls, respectively. To verify whether the complex formed by YTHDC1 and DDX5 is dependent on the presence of RNA, we performed DDX5 immunoprecipitation upon treatment with RNase A (Supplementary Fig. 4b). As shown in Supplementary Fig. 4c, the presence of YTHDC1 in the DDX5 IP fraction was maintained after RNA degradation.

To further investigate the functional relationship between YTHDC1 and DDX5, we addressed their involvement in pathways regulating RMS proliferation by depleting these factors, either individually or together, through a siRNA-based approach in RD cells (Supplementary Fig. 4d). FACS analysis showed that cells depleted for YTHDC1 or DDX5 experienced a block in the G2/M phase transition, which was stronger when the two factors were knocked-down in combination (Supplementary Fig. 4e).

Previous studies reported the activity of DDX5 on genes involved in cell cycle control^40^. We then tested some of these targets (CCND1 and C-MYC) but we could not find any alteration of their expression (Supplementary Fig. 4f). Therefore, it is possible that DDX5 downregulation in our system affects a different set of targets.

Lastly, in order to test whether YTHDC1 and DDX5 expression correlates in other cellular systems, we retrieved their expression levels from a human database, which collects over 2000 human cancer samples^41^, and found a statistically significant positive correlation between the two genes (Supplementary Fig. 4g). The correlation is also conserved in mouse, as reported by two different databases: COXPRESdb^42^, where genes are ranked according to their degree of co-expression to a gene under analysis (Supplementary Fig. 4h, left panel), and FNTM^43^ which predicts functional relationships between proteins across multiple tissues (Supplementary Fig. 4h, right panel). In the latter case, we focused our analysis on skeletal muscle, where we detected YTHDC1 in the network of the top DDX5 functionally related genes.

Given the close relationship of DDX5 with YTDHC1, already known for its role in the biogenesis of circRNAs, we tested its ability to participate in such process. Therefore, we depleted DDX5 with siRNAs in RD and RH4 cells (Supplementary Fig. 4i) and performed total RNA-seq. Differential expression analysis identified a dramatic downregulation of circRNAs upon DDX5 knock-down (410 in RD and 438 in RH4, corresponding to 83% and 91% of the deregulated species, respectively), with only few cases of upregulation (85 in RD = 17% of deregulated; 43 in RH4 = 9% of deregulated; Fig. 4e, f, Supplementary Data 1). The same trend was also observed when analyzing the “*high-confidence*” group of circRNAs (Fig. 4f and Supplementary Fig. 4j).

Similarly to what was previously observed for YTHDC1 depletion and even with a stronger magnitude, this decrease resulted highly specific for circRNAs, as witnessed by the lack of enrichment of linear RNAs (Fig. 4f) and by the fact that affected circRNAs mainly belonged to the “*discordant*” downregulated class (Fig. 4g, Supplementary Fig. 4k, l). These results demonstrate that DDX5 is a regulator of circRNA accumulation levels in RMS.

### DDX5 and YTHDC1 directly regulate a subset of circRNAs promoting their upregulation in RMS

The analogous effects of YTHDC1 and DDX5 depletion on circRNA accumulation suggested that they might regulate a common set of targets. To investigate their involvement in circRNA biogenesis and exclude transcriptional modulation, we focused only on circRNAs found as “*discordant*” in the depletion of these two factors.

**Fig. 5:**
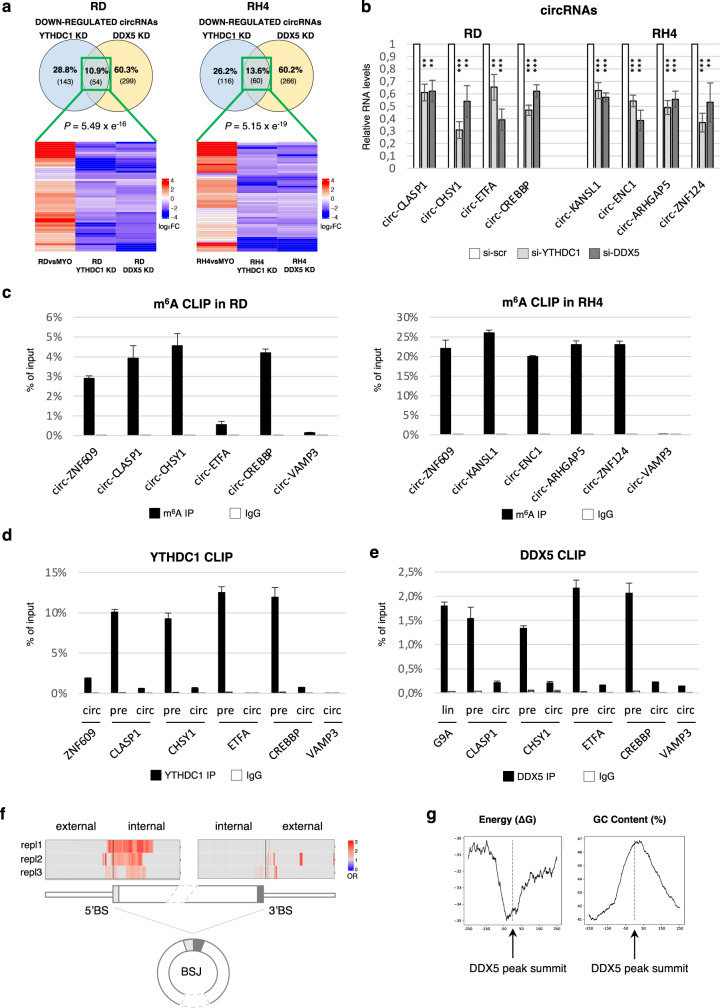
DDX5 and YTHDC1 directly regulate a subset of circRNAs promoting their upregulation in RMS. **a** Venn diagrams (upper panels) showing the overlap between discordant circRNAs downregulated upon YTHDC1 knock-down and those downregulated upon DDX5 knock-down, either in RD (left panel) or in RH4 (right panel). Significance was calculated via Fisher exact two-tailed test. Heatmaps (lower panels) showing log_2_ fold change of circRNAs at the overlap of the upper Venn diagrams in the comparison between each RMS cell line and wild-type myoblasts as well as in YTHDC1 or DDX5 knock-down in the respective RMS line. **b** Relative RNA levels of selected circRNAs upon YTHDC1 knock-down (“si-YTHDC1”) or DDX5 knock-down (“si-DDX5”) in RD or RH4. Values are normalized against GAPDH and expressed as relative quantity with respect to scramble siRNA treatment (“si-scr”) set to a value of 1. The relative RNA quantity in the bars is represented as mean of the fold change with standard deviation. *n* = 3 biologically independent replicates. The *ratio* of each sample *versus* its experimental control was tested by two-tailed Student’s *t* test with correction for multiple test comparison (FDR Benjamini-Hochberg). * indicates a test-derived *p* value < 0.05, ** indicate a *p*-value < 0.01, and *** a *p* value < 0.001. **c** Levels of selected circRNAs recovered from a representative m^6^A CLIP in RD (left panel) and RH4 (right panel). CircZNF609 and circVAMP3 were used as positive and negative controls, respectively; immunoprecipitation with IgG was used as control. **r**, Levels of selected circRNAs recovered from a representative m^6^A CLIP in RH4 either in control condition (“si-scr”) or upon METTL3 (“si-METTL3”) or DDX5 knock-down (“si-DDX5”); immunoprecipitation with IgG was used as control. The relative RNA quantity in the bars is represented as mean of technical replicates with standard deviation. *n* = 2 biologically independent replicates. **d**, **e** Levels of precursors or mature circRNAs recovered from a representative YTHDC1 (**d**) or DDX5 (**e**) CLIP experiment. Values are expressed as percentage of input with standard deviation. CircZNF609 (**d**) and G9A (**e**) were used as positive controls. CircVAMP3 was used as negative control. The relative RNA quantity in the bars is represented as mean of technical replicates with standard deviation. n = 3 biologically independent replicates. **f** Heatmap representing DDX5 binding enrichment in meta-BSJ proximal regions comparing the set of “*high-confidence*” downregulated circRNAs upon DDX5 depletion in RH4 cells with a set of selected controls from invariant circRNAs. For each DDX5 RIP-seq replicate (“Repl1-2-3”), the odds *ratio* (“OR”) related to each 100nt window analyzed is depicted. Only bins with significant odds *ratio* (*p* value < 0.05) were colored. Statistical significance was assessed using two-sided Fisher exact test. **g** Line plots representing ∆G (left panel) and GC content (right panel) of 500nt regions centered to DDX5 peak summit of RIP-Seq replicate 1 (see Supplementary Fig. 5 v for replicate 2 and 3). Source data are provided as a Source Data file.

Venn diagrams in Fig. 5a (upper panel) highlighted a statistically significant overlap for the circRNAs downregulated upon both DDX5 and YTHDC1 knock-down in RD (10.9%) as well as in RH4 cells (13.6%). Interestingly, among these species the vast majority showed increased expression in RMS if compared to wild-type myoblasts, confirming that YTHDC1 and DDX5 are able to sustain the expression of a specific class of circRNAs in the tumor (heatmaps in Fig. 5a, lower panel). It is noteworthy that when considering only the circRNAs affected by YTHDC1, 27% in RD and 34% in RH4 were also regulated by DDX5, thus indicating that a considerable fraction of the circRNAs whose biogenesis depends on YTHDC1 also requires DDX5. In order to validate RNA-seq results, we selected a restricted number of circRNA candidates in both cell lines according to their expression levels. After verifying by qRT-PCR the specific amplification and the resistance of such molecules to RNase R treatment (Supplementary Fig. 5a, b), we confirmed that the downregulation was specific for the circular isoforms (Fig. 5b) while the linear counterparts were overall unaffected (Supplementary Fig. 5c). Importantly, we verified that the downregulation of YTHDC1 or DDX5 did not reduce the levels of the interacting partner at the RNA and protein levels (Supplementary Fig. 5d, e). In fact, we observed that the levels of DDX5 did not change upon YTHDC1 depletion, while even a variable increase of YTHDC1 was detected upon DDX5 knock-down.

Given that the tested set of circRNAs was affected by the depletion of YTHDC1 or DDX5 proteins, we evaluated their responsiveness to a combined knock-down of both factors. Even if the trend was conserved in the double knock-down, we did not observe a strong additive effect which would suggest a cooperative activity (Supplementary Fig. 5f).

Previous studies reported that YTHDC1 and the RNA helicases UAP56 or URH49 can participate in circRNA nuclear export^44,45^. In order to rule out that the reduced circRNA expression observed upon YTHDC1 and DDX5 knock-down was due to nuclear retention, we performed cell fractionation assays in RD and RH4 cells: none of the tested circRNAs showed any mis-localization (Supplementary Fig. 5g, h).

Furthermore, even though YTHDC1 and DDX5^46,47^ are both nuclear factors, we ruled out that the observed reduction of circRNAs could be related to their reduced stability by performing actinomycin D treatment in RD cells. As expected, besides the high stability of circRNAs over time (up to 12 hrs), we did not observe reduced half-life either upon YTHDC1 nor in DDX5 depletion (Supplementary Fig. 5i). Altogether, these data support a role of these two factors in promoting circRNA biogenesis.

Finally, we also addressed the methylation status of the mature circRNAs through m^6^A CLIP assays in RMS lines. We found that the species analyzed were enriched in the immunoprecipitated fraction if compared to circZNF609 and circVAMP3 used as positive or negative control, respectively^25,48^, supporting their m^6^A-mediated production (Fig. 5c).

In accordance with the hypothesis that YTHDC1/DDX5 promote back-splicing, CLIP analysis in RD cells (Supplementary Fig. 5j) revealed their direct binding to circRNA precursors: all the tested precursor species were immunoprecipitated at levels comparable with the positive controls and higher than the negative control circVAMP3, whose expression was not affected by the knock-down of either protein (Fig. 5d, e).

At the same time, in line with YTHDC1 and DDX5 activity being exerted on the precursor RNA, the enrichment of these factors on mature circRNA molecules was lower (Fig. 5d, e).

In order to further corroborate these observations, we analyzed DDX5 RIP-seq data performed in RH30, a cell line resembling the ARMS subtype^35^(Supplementary Fig. 5k). We observed enriched binding of DDX5 at the *loci* encoding for circRNAs downregulated upon DDX5 or YTHDC1 knock-down, (Supplementary Fig. 5l). Notably, such enrichment was even more pronounced in “*high-confidence*” circRNAs (Supplementary Fig. 5m). Moreover, when more stringent *p*-value cutoffs were applied in order to select differentially expressed circRNAs, the binding increased only for the downregulated species, confirming the specificity of DDX5 activity on this category (Supplementary Fig. 5n). Examples of DDX5 binding on validated circRNAs are reported in Supplementary Fig. 5o.

Interestingly, despite RIP-seq does not allow an accurate definition of binding sites, we found that DDX5 binding is enriched in the exonic regions proximal to the BSJ, mainly in the upstream 5’ splice site, of downregulated circRNAs if compared to a control set of invariant ones, even when considering multi-exonic circRNAs (Fig. 5f, Supplementary Fig. 5p).

In consideration of DDX5 activity as RNA helicase, we used RNAfold to predict the propensity of regions bound by DDX5 to form secondary structures. Each binding site was located inside a window of 500 nt with the most intense signal located in the center. We observed a decreased ∆G corresponding to these positions; moreover, when analyzing the GC content of DDX5 binding sites we found a clear peak again in the middle of the window (Fig. 5g, Supplementary Fig. 5q). Altogether, these data suggest that DDX5 binding sites fall in potentially structured regions. To test whether DDX5 helicase activity in these regions is required to expose target sequences to m^6^A modification, we performed m^6^A immunoprecipitation in control or DDX5 knock-down conditions. As a control, we conducted the same experiment upon METTL3 depletion. As shown in Supplementary Fig. 5r, the recovery of methylated circRNAs decreased upon METTL3 downregulation, while it was unaffected by DDX5 depletion, allowing us to conclude that DDX5 is not required for global m^6^A deposition.

## Discussion

To date, many different functions have been ascribed to circRNAs: from the control of alternative splicing and transcription in the nucleus, to miRNA and protein sponging activity in the cytoplasm as well as the ability to encode for functional peptides and to control mRNA protein synthesis^1,27^. Moreover, mounting evidence indicates the close association of circRNAs with the onset and progression of pathological conditions, including cardiovascular and neurodegenerative diseases^9,49,50^, metabolic disorders^51^, and cancer^52^.

An interesting case is represented by circZNF609, which is upregulated in RMS biopsies and cell lines and acts as a crucial regulator of cancer growth: indeed, the downregulation of this circRNA, and not of its linear counterpart, strongly reduced the proliferation rate of in vitro cultured myoblasts and of RMS cell lines^26,53^. Interestingly circZNF609 was shown to control cell proliferation through the regulation of CKAP5 protein levels, and in turn the dynamic functions of the mitotic apparatus^27^. This mechanism resulted quite relevant when considering that this RNA is upregulated in several tumors and it can justify its role in many different tumorigenic conditions^48,54–59^.

Besides circZNF609, the activity of other onco-circRNAs was often linked to their dysregulated levels, even though the molecular mechanisms driving such alterations are unknown in most cases. Since circRNAs originate from a non-conventional splicing event which is alternative to that of the linear counterpart, it is compelling to investigate whether changes of their expression could be attributed to altered biogenesis and not merely to transcriptional control.

In this work, through a genome-wide approach, we unveiled the existence of factors able to promote the production of a defined subset of circRNAs in RMS, independently from their linear counterparts. RNA-seq analysis indicated that, in comparison with linear RNAs, a much smaller fraction of circRNAs was commonly expressed between control myoblasts and RMS cell lines, suggesting that specific pathways operate on circRNAs to modulate and diversify their expression in the tumor. Nonetheless, in line with the fact that the two tumor cell lines represent subtypes of the same tumor, they shared a higher number of common circRNAs with respect to the healthy control.

Differential expression analysis comparing each RMS cell line with control myoblasts revealed no preferential modulation for linear RNAs, whereas circRNAs predominantly exhibited upregulation, implying the existence of mechanisms specifically sustaining their biogenesis in the tumor. In agreement with this hypothesis, expression levels of circular and linear isoforms originating from the same gene indicated that the upregulation of circRNAs in most cases was not the mere result of transcriptional activation of the *locus*.

We previously demonstrated that m^6^A, a key player for the occurrence and development of several tumors^60^, is able to favor back-splicing^19^. Starting from the observation that m^6^A regulators were increased in RMS tumors and cell lines when compared to healthy conditions, we investigated whether the m^6^A machinery could be responsible for the promotion of circRNA expression. Indeed, we identified the reader YTHDC1 as one of the links between m^6^A modification and altered circRNA biogenesis in the tumor.

Moreover, reinforcing the functional dependency of RMS tumorigenicity on the presence of m^6^A modification, we showed that depletion of MACOM complex components strongly reduced RMS cells proliferation and migration rate, two biological processes often promoted by the activity of onco-circular RNA molecules^52^.

It is noteworthy to mention that back-splicing is a form of alternative splicing (AS). AS is a finely tuned process, where different outcomes are intimately dependent on the crosstalk of specific regulatory RNA binding proteins and splicing factors.

With a view to extending the network of YTHDC1 partners, we identified DDX5 as a mediator of the back-splicing reaction. This factor is an important regulator of RNA splicing and its levels are particularly high in RMS patients and cell lines. Moreover, DDX5 was found to interact with YTHDC1 supporting the idea that they can work in a complex. Indeed, not only a significant set of circRNAs was regulated by both factors in RMS, but we also observed an overlap between transcripts enriched for their binding, further reinforcing the hypothesis that they can act on a common pathway.

Even if DDX5 knock-down did not affect the overall m^6^A content of the tested circRNAs, it is possible that DDX5 could operate by making specific neighboring m^6^A target sites available for modification and for YTHDC1 binding. This hypothesis cannot be ruled out since it is challenging to distinguish local m^6^A perturbations important for the back-splicing reaction from the ones used for the regulation of linear splicing. On the other hand, it is also possible that the two factors act independently, with the helicase operating by exposing sequences important to promote the back-splicing reaction and YTHDC1 by recruiting specific splicing factors.

Altogether, our data indicate a pathway where the biogenesis of a class of circRNAs relies on the interplay between the m^6^A reader YTHDC1 and the DDX5 helicase. Notably, the coupled increase of YTHDC1 and DDX5 expression in a larger set of human cancers suggests that both factors might be relevant for the biogenesis of specific subsets of circRNAs in tumors other than RMS.

Considering that the depletion of YTHDC1/DDX5 reduces cell proliferation and that many circRNAs are involved in the control of cell growth, we speculate that the modulation exerted by YTHDC1 and DDX5 may act as an oncogenic feature in this tumor.

Future work will allow establishing how the upregulation of specific circRNAs correlates with tumor onset and progression; moreover, the inhibition of the m^6^A machinery could become an interesting therapeutic approach similar to what is suggested for the treatment of other pathologies, such as AML^60^. Finally, such distinctive circRNA repertoire might also represent an important reference dataset in order to identify candidates to be used as diagnostic biomarkers for the identification of the tumor subtype in RMS patients.

## Methods

### Cell culture

Wild-type human primary myoblasts (Telethon Biobank) were obtained from a skeletal muscle biopsy from a 2-year-old male child. No information available about their authentication. They were cultured in growth medium (GM): DMEM high glucose (Sigma-Aldrich, Saint Louis, MO, USA), 10% FBS (Sigma-Aldrich), l-glutammine (Sigma-Aldrich) 2 mM, insulin (Sigma-Aldrich) 50 mg/ml, FGFb (Millipore-Merck) 25 ng/ml, EGF (Corning, Corning, NY, USA) 1 ng/ml, penicillin-streptomycin 1× (Sigma-Aldrich). Human ERMS RD cell line (embryonal rhabdomyosarcoma cell line from a female patient) and ARMS RH4 cells line (alveolar rhabdomyosarcoma cell line from a female patient) cells were cultured in DMEM high glucose (Sigma-Aldrich) supplemented with 10% FBS (Sigma-Aldrich), l-glutammine (Sigma-Aldrich) 2 mM and penicillin-streptomycin 1× (Sigma-Aldrich). All cells were cultured at 37 °C in a humidified atmosphere of 5% CO2. All cell lines were tested for mycoplasma contamination.

### Cell transfection

Cells (150–200 × 10^3^) were plated in 35 mm plates and transfected 12 hrs later with the siRNA against the target selected—except for METTL3, where a mix of four siRNAs was used—or the negative control (final concentration 30 nM) using 5 μl of Lipofectamine RNAiMAX Reagent (Thermo Fisher Scientific) and 300 μl of Opti-MEM (Thermo Fisher Scientific). The medium was replaced 5–12 hrs later. Cells were harvested 48 hrs later or used for further analyses. For FACS analysis, cells (600 × 10^3^) were plated in 60 mm plates and transfected 12 hrs later with the siRNA against the target selected or the negative control (final concentration 30 nM) using 10 μl of Lipofectamine RNAiMAX Reagent (Thermo Fisher Scientific) and 600 μl of Opti-MEM (Thermo Fisher Scientific). Cells were passed 5 hrs later to a 100 mm plate and collected 48 hrs later. For the double knock-down of YTHDC1 and DDX5, a combination of two different siRNA was used at a final concentration of 60 nM. Cells were passed 5 hrs later to a 100 mm plate and collected 72 hrs later. For actinomycin D treatment, cells depleted for YTHDC1 and DDX5 were split into two different plates and, after 12 hrs, harvested or kept in their medium added with actinomycin D (5 mg/ml, Sigma-Aldrich) for 6 hrs or 12 hrs.

### Protein analysis

Cells were harvested in a suitable volume of Protein Extraction Buffer (Tris pH 7.5 100 mM, EDTA 1 mM, SDS 2%, PIC1X (Complete, EDTA free, Roche) and incubated 10 min on ice, then incubated on a rotator for 30 min at 4 °C and centrifuged at 16000 × *g* for 10 min at 4 °C. The supernatant was transferred to a clean tube, used for subsequent analyses, or stored at −80 °C. Total protein concentration was measured through the Bradford reagent (Bio-Rad Protein Assay) following manufacturer’s instructions.

20–50 μg of proteins were loaded on 4–12% bis-tris polyacrylamide gel (Thermo Fisher Scientific) and transferred to a nitrocellulose membrane. The membrane was blocked in 5% milk and then hybridized with specific antibodies for 1 hr at room temperature or overnight at 4 °C. After three washes in TBST, the filter was hybridized with the corresponding secondary antibody, if required, for one hour at room temperature. All antibodies used in this study are reported below.

Protein detection was carried out with WesternBright® ECL Chemiluminescent HRP Substrate (Advansta) or with Clarity Max Western ECL Substrate (Bio-Rad). Images were acquired using a ChemiDoc^TM^ MP Imager (Bio-Rad) and images were analyzed using Image Lab^TM^ 5.2.1 Software (Bio-Rad).

### RNA isolation and analysis

Cell fractionation assays were carried out using the PARIS^TM^ Kit (Thermo Fisher Scientific) according to the manufacturer’s protocol. Nuclear, cytoplasmic, or total RNA in this study was extracted with Qiazol reagent (QIAGEN) and Direct-zol RNA Miniprep (Zymo Research) kit according to the manufacturer’s specifications. For immunoprecipitation experiments, the RNA was recovered through standard phenol-chloroform extraction and precipitation. When needed, DNAse I treatment was performed (Thermo Fisher Scientific).

Reverse transcription reactions for routine experiments were performed using PrimeScript RT Master Mix (Takara Bio), while for RNA derived from CLIP experiments the SuperScript VILO cDNA Synthesis Kit (ThermoFisher Scientific) was used, according to manufacturer’s protocol. RT-qPCR analyses were performed using PowerUp SYBR Green Master Mix reagent (ThermoFisher Scientific), according to the manufacturer’s instructions. DNA amplification was monitored on an Applied Biosystems^TM^ 7500 Fast or StepOnePlus System qPCR instrument with the 7500 Software (Applied Biosystems) version 2.3 or with the StepOneTM Software (Applied Biosystems) version 2.3, respectively.

Relative RNA quantity was calculated as the fold change (2^−ΔΔCt^) with respect to the experimental control sample set as 1 and normalized over ACTN1 or GAPDH mRNA, or to an external spike-in when needed. A complete list of the oligonucleotides used for qRT-PCR experiments is provided below.

For RNase R treatment, 3 μg of total RNA were diluted in 20 μL reaction with 5 U of RNase R (Epicenter), then incubated 15 min at 37 °C. We purified the digested RNA added with 4 pg of a DNA spike-in molecule for qPCR normalization by phenol-chloroform extraction. DNA spike-in was produced as described by Legnini et al. 2017^53^.

### m^6^A CLIP

m^6^A CLIP was performed according to the protocol described by Linder et al.^61^ with some modifications. Briefly, total DNase I-treated RNA was purified from RD or RH4 cells, 20 μg of RNA were diluted in IP buffer supplemented with RNase Inhibitor (Thermo Fisher Scientific) and incubated with 5 μg of anti-m^6^A antibody or IgG for 2 hrs at 4 °C rotating head over tail and crosslinked, 10% of the solution was saved to be used as input, the leftover incubated with protein A/protein G Dynabeads (Thermo Fisher Scientific) for 2 hrs at 4 °C. Bead-bound antibody-RNA complexes were washed and recovered. After phenol-chloroform extraction and precipitation, RNA was resuspended in 30 μl, and 7 μl were reverse-transcribed with VILO cDNA Synthesis Kit (ThermoFisher Scientific) in a 10 μl reaction. RT-qPCR was performed to evaluate targets enrichment.

### CLIP assay

150 mm plates with RD cells at maximum 80% confluency were washed twice with ice-cold PBS 1× (Sigma- Aldrich) and irradiated with 0.4 J/cm^2^ of 254 nm UV light. Cells were lysed in RIPA buffer (Tris-HCl pH 8 20 mM, NaCl 100 mM, EDTA 0.5 mM, NP-40 0.5%, SDS 0,1%) supplemented with PIC 1× and RNase Inhibitor (Thermo Fisher Scientific). Lysates were incubated on ice for 15 min at 4 °C, then passed through a 21 G needle. Lysates were spun down at 16000 × *g* for 10 min at 4 °C and the supernatants were collected, then quantified with Bradord assay. Lysates were pre-cleared for 30 min on a rotator at 4 °C with protein A Dynabeads (Thermo Fisher Scientific). 10% of the lysate was saved to be used as input, while for each immunoprecipitation 1 mg of extract was incubated with 1 μg of specific or IgG antibody overnight on a rotator at 4 °C. The next day, 50 μl of pre-washed protein A Dynabeads were added to the samples and incubated on a rotator for 2 hrs at 4 °C. Bead-bound antibody-RNA complexes were recovered on a magnetic rack, washed three times on a rotator for 2 min at room temperature with 500 μl Wash Buffer (Tris-HCl pH 7.4 50 mM, NaCl 150 mM, MgCl_2_ 1 mM, NP-40 0.05%) and three times with High-Salt Wash Buffer (Tris-HCl pH 7.4 50 mM, NaCl 500 mM, MgCl2 1 mM, NP-40 0.05%). One fifth of each sample was used for protein analysis, while 4/5 were used for RNA analysis after 1 hr treatment with 10 μl Proteinase K (Roche) at 70 °C in 90 μl PNK buffer (Tris-HCl pH 7.4 10 mM, NaCl 100 mM, EDTA 1 mM, SDS 0.5%). After reverse-transcription of the extracted RNA with VILO cDNA Synthesis Kit (ThermoFisher Scientific), RT-qPCR was performed to evaluate targets enrichment.

### Cell count assay

RD or RH4 cells transfected with siRNA for METTL3 or with negative control in a 35 mm plate were passed after 5 hrs to a 60 mm plate. 48 hrs later, cell proliferation was evaluated by counting trypsinized cultures. Relative number of cells was calculated as fold change with respect to the experimental control sample set as 1.

### Flow cytometry analysis of cell cycle

Cells were trypsinized and counted. An equal number of cells for each experimental condition was used for the analysis. Cells were washed once with PBS 1× (Sigma-Aldrich), fixed in 2 mL ice-cold 70% ethanol per 1 × 10^6^ cells, and incubated at 4 °C overnight. Then cells were centrifuged for 5 min at 300 × *g* at 4 °C, washed once with PBS 1× (Sigma-Aldrich), and pelleted again. Cells were then resuspended in 300 μL PBS (Sigma- Aldrich) supplemented 100 μg/ml RNase A (Qiagen) and 50 μg/ml Propidium Iodide (Sigma-Aldrich), and then incubated in the dark for 30 minutes at room temperature. Samples were processed using a using a BD LSRFortessa (BD Biosciences, San Jose, CA, USA). The percentages of cells in different phases of the cell cycle were determined using the FlowJo V9.3.2 computer software (TreeStar, Ashland, OR, USA). At least 10 × 10^3^ events for each sample were acquired.

### Transwell-migration assay

48 hrs after transfection, cells were trypsinized and counted. 150 × 10^3^ cells were pelleted for 5 min at 153 × *g* and resuspended in 600 μl serum-free medium, then seeded in the transwell inserts (Greiner Bio-One). Inserts were put in 35 mm plates and 900 μl complete medium was added outside the insert. Cells were left at 37 °C in a humidified atmosphere of 5% CO_2_ for 15 hrs to migrate. After that, medium was removed and cells were fixed with 1 ml ice-cold 4% paraformaldehyde in PBS 1× (Sigma-Aldrich) in for 15 min at 4 °C. Cells were washed twice with 1 ml PBS 1× (Sigma-Aldrich) and then incubated for 20-40 min in a 1 ml solution with 1 μg/ml DAPI (Sigma-Aldrich) and 0.5% Triton^TM^ X-100 (Sigma-Aldrich) diluted in PBS 1× (Sigma-Aldrich). Cells were then washed three times with PBS 1× (Sigma-Aldrich). Samples were imaged using an inverted microscope Zeiss Axio Observer A1 Phase Contrast supported with Zeiss AxioCam MRm camera. Images were acquired using a Zeiss Plan-Neofluar ×10 objective (NA 0.3) and were collected and analyzed with the AuxioVision software (Zeiss) version 4.8.2. For each condition, 50–70 fields were acquired and analyzed. The analysis of images was performed with the ImageJ software^62^. After applying proper thresholding on image, DAPI signals were counted using the ImageJ tool “Analyze Particles”.

Relative number of migrated cells was calculated as fold change with respect to the experimental control sample set as 1.

### Cell fractionation

48 hrs after transfection, cells were fractionated by the Ambion PARIS Kit (Life Technologies), according to manifacturer’s instructions. After RNA extraction, for each condition 600 ng of cytoplasmic RNA and an equal volume of nuclear RNA fraction were reverse-transcribed and analyzed by RT-qPCR. An external RNA spike- in RNA was added before RNA extraction and later used as control for normalization.

### Co-immunoprecipitation

RD cells were washed twice with ice-cold PBS (Sigma-Aldrich), gently scraped with ice-cold PBS (Sigma- Aldrich), and pelleted at 153 × *g* for 5 min at 4 °C. Cell pellets were lysed in Lysis buffer (50 mM Tris-HCl pH 7.5, 150 mM NaCl, 1 mM EDTA, 1% NP-40, 0.1% SDS) supplemented with PIC (Roche) 1×. Lysates were incubated on a rotator for 30 min at 4 °C, then passed through a 21 G needle. Lysates were spun down at 16000 × *g* for 10 min at 4 °C and the supernatants were collected. 600–1500 μg of extract were quantified through a Bradford assay, diluted in 500–1000 μl of co-IP buffer (50 mM Tris-HCl pH 7.5, 150 mM NaCl, 1 mM EDTA, 0.25% NP-40, 5% glycerol) supplemented with PIC (Roche) 1× and incubated with 750 ng–5 μg of primary antibody or control IgG overnight at 4 °C on a rotator. 10% of the extract was saved to be used as input. The next day, 50 μl Dynabeads Protein G (for DDX5 IP) or Dynabeads Protein A (for YTHDC1 IP, Thermo Fisher Scientific) pre-washed three times with co-IP buffer were added to the samples. Samples were then incubated on a rotator for 4 hrs at 4 °C. The beads were recovered through a magnetic rack and washed four times for 5 min on a rotator at room temperature with 500 μl co-IP buffer. Beads were then resuspended in 100 μl co-IP buffer and transferred to clean tubes. Beads were then recovered on a magnetic bead, resuspended in Lysis buffer, LDS 1× (Biorad) supplemented with 1 mM DTT, and heated for 10 min at 90 °C. Variable fractions of input and IP or IgG samples were loaded for western blot analysis.

For the co-immunoprecipitation experiments with RNase A treatment, lysates were incubated with 1 mg/ml RNase A (Qiagen) for 30 min at room temperature on a rotator before proceeding with the antibody incubation. A fraction of the lysate was saved after RNase A treatment for total RNA extraction and control of the treatment efficacy, which was performed by loading equal amounts of treated or untreated RNA on 1% agarose gel.

### RNA-seq

For the sequencing of human wild-type myoblasts, RD cells or RH4 cells either in control conditions (si-SCR) or depleted for YTHDC1 or DDX5 total RNA was extracted from three biological replicates. The RNA library for all samples was produced using Stranded Total RNA Prep with Ribo-Zero Plus (Illumina). All samples were sequenced on an Illumina Novaseq 6000 Sequencing system with an average of about 50 million 150 nucleotides long paired-end read pairs.

### CircRNAs detection and differential expression analysis

Trimmomatic^63^ (v0.39) and Cutadapt^64^ (v3.2) were used to remove adapter sequences and poor-quality bases; the minimum read length after trimming was set to 35. Reads aligning to rRNAs were filtered out; this first alignment was performed using Bowtie2 software (v2.4.2) (https://bowtie-bio.sourceforge.net/bowtie2/index.shtml).

Reads were then aligned to the human reference genome (GRCh38) using BWA-MEM^65^ (v0.7.17) with -T 19 option. CircRNA detection in each sample was then carried out using CIRI2 software^28^(v2.0.6), which is able to identify circRNAs by searching for reads that map to back-splicing junctions. To identify circRNA host genes, the program was provided with Ensembl 99 gene annotation^66^. For each back-splicing event found, CIRI2 reports the number of reads mapping to the back-splicing junction and on the corresponding linear splicing junctions, calculated summing all the reads mapping linearly on both the splice junctions involved in back-splicing; the latter are not reported if no read is assigned to the back-splicing junction, even if the circular RNA is detected in other samples. In order to count the reads mapping to linear splicing sites in samples in which no reads were mapped to corresponding back-splicing junctions detected in other samples, alignment files from each sample were modified by adding reads mapping to circRNAs found only in other samples and CIRI2 was rerun on these files. Alignments files operations were performed using Picard suite (v2.24.1) (https://broadinstitute.github.io/picard/) and SAMtools (v1.10) (http://www.htslib.org).

To evaluate the differential expression of circRNAs between knock-down and si-scramble conditions, we provided the edgeR R package^67^(v3.34.1) with the read counts of both the back-splicing events and the linear splicing events detected. Events not having 2 or more counts in at least three samples were not tested for differential expression. Since the average number of junctions (linear and back-splicing) identified as expressed in each contrast was 33139 we assumed that most of them were not differentially expressed and then normalized samples of each contrast using standard edgeR normalization (TMM); model fitting and testing was performed using the glmFIT and glmLRT functions.

Reads mapping to back-splicing junctions and to their cognate linear splicing junctions were converted to Count Per Million (CPM) values using edgeR and used for circRNA and linear RNA quantification Given the low number of reads used for testing, we decided to use *p*-value instead of false discovery rate to select for differentially expressed events, setting the significance threshold value to 0.05.

For the analyses of linear RNAs in Supplementary Fig.1a (right panel) reads were aligned to the human reference genome (GRCh38) using STAR^68^ (v2.7.7a) and counts were retrieved using HTSeq software^69^ (v0.13.5). In this analysis, circRNAs with at least 2 counts in at least 2 samples and linear RNAs with at least 5 counts in at least 3 samples in each condition were taken into consideration. “*High-confidence*” circRNAs were identified through three software: CIRI2, DCC^30^ (v0.5.0), and circ^29^ (v2.3.8).

CircRNAs identification with DCC software was performed following the standard workflow for paired-end sequencing specifying these parameters: -ss -Pi -fg -Nr 2 3 -D -G; circExplorer2 analysis was performed using the one-command pipeline.

For both tools reads alignment to the reference genome was performed using STAR with parameters suggested by each program. For both tools, circRNAs not having 2 or more counts in at least three samples in each contrast were excluded as not expressed.

CircRNA exon number was defined considering a representative transcript for each associated locus. In case of ambiguous reference, where the circRNA region overlapped multiple isoforms, the representative transcript was selected prioritizing the isoform with the same biotype of the gene, with BSJ boundaries strictly coincident with exon junctions, minimizing the number of exons included and the first exon included in the circRNA labeled with the lowest number.

### DDX5-binding sites analysis

DDX5 narrow peaks files related to DDX5 RIP-Seq performed in RH30 cell line were retrieved from GEO record GSM5333799^35^. In order to consider the most similar system to RH30, DDX5 binding analyses were performed in RH4 cell line.

For the analysis of DDX5 circRNA interactors showed in Supplementary Fig. 5, DDX5 significant peaks were intersected with back-splicing genomic coordinates using bedtools suite^70^ (v2.29.1). CircRNAs with at least one peak in two DDX5 RIP samples were defined as DDX5 interactors.

For meta-BSJ enrichment analysis, we selected “high-confidence” downregulated circRNAs upon DDX5 depletion. For each circRNA, we analyzed DDX5 binding enrichment considering a 100nt sliding window (with 10nt progression step) in a region from 1000nt upstream to 1000nt downstream of each extremity of the BSJ (5’ and 3’ splice sites). For each window 2 control regions were selected from invariant circRNAs considering the same relative position from the BSJ. The most similar controls were selected taking into consideration the properties of the window (% of nucleotides overlapping exons, 5’UTR, CDS, and 3’UTR regions) and the circRNA features (host gene biotype and circRNAs exon number). We intersected circRNAs windows with DDX5 peaks using bedtools suite^71^ and we compared the number of regions containing or not a DDX5 peak among the downregulated set and the control set using Fisher’s exact test. With the same methodology, we checked that the properties of the windows previously described were balanced between the two sets.

DDX5 binding enrichment in every exon and intron of biexonic, three-exonic, and multi-exonic circRNAs was assessed considering the structure of representative isoforms selected as described in the “CircRNAs detection and differential expression analysis“ section. Only the “high-confidence” circRNAs with BSJ boundaries strictly coincident with exon junctions were evaluated. Exons and introns most proximal to 5’ back-splicing were defined as “Ex_A” and “Intr_A” while those proximal to the 3’ back-splicing were defined as “Intr Z” and “Ex Z”, the other medial regions were defined as “Ex_M” and “Intr_M”. For each region type of the three groups (bi-exonic, three-exonic and multi-exonic group), DDX5 enrichment was evaluated comparing the number of regions containing or not a DDX5 peak among the downregulated and invariant circRNAs using Fisher’s exact test.

Peaks related to high-confidence downregulated circRNAs were retrieved using bedtools intersect. When peaks overlap each other, the longest peak was selected. Considering this set of peaks, we generated a window of 500nt with the most intense signal of DDX5 binding in the center (peak summit). For each position in the window, sequences of a region from 75nt upstream and 75nt downstream the position were retrieved using *bedtools getfasta* assigning the strand of the associated circRNA. Then, RNAfold algorithm^72^ (v2.4.17) was used to predict ∆G. Coverage data of RIP-seq IP samples were normalized on Input using bamCompare function from deepTools (v3.5.1) (https://deeptools.readthedocs.io/en/develop/). IGV software (v2.11.9) was used for DDX5 peaks visualization in Supplementary Fig. 5o^73^. Heatmaps graphical representations were depicted using ComplexHeatmap R package (v2.8.0) (https://bioconductor.org/packages/release/bioc/html/ComplexHeatmap.html).

### Quantification and statistical analysis

The distribution and deviation of data shown in the figures of this work, the statistical tests used to calculate significant differences, and the exact value of n (e.g., the number of biological replicates of the experiments) are denoted in figure legends. In figure legends “SD” stands for “standard deviation” and “SEM” stands for “standard error mean”. Significance values were depicted in the figures using the following key legend: *p < 0.05, **p < 0.01, ***p < 0.001. In the box plots interquartile range spans from 75th percentile and 25th percentile of data with the median indicated as line in the box (50th percentile). Upper whisker indicates values larger than the 75th percentile within 1.5 times interquartile range. Lower whisker indicates values smaller than the 25th percentile within 1.5 times interquartile range. Outside values are >1.5 times and <3 times the interquartile range beyond either end of the box. When needed, data were further processed and analyzed with Microsoft Excel version 16.71.

### Oligonucleotides used in this study

The oligonucleotides used for this study are listed in Supplementary Table 1.

### Target sequence of siRNAs used in this study

siRNAs used for this study are listed (sense sequences) in Supplementary Table 2.

### Antibodies used in this study

The antibodies used for this study are listed in Supplementary Table 3.

### Reporting summary

Further information on research design is available in the Nature Portfolio Reporting Summary linked to this article.

## Supplementary information

Supplementary Information

Description of Additional Supplementary Files

Supplementary Data 1

Reporting Summary

## Data Availability

All data supporting the findings of this study are available within the paper and its Supplementary Information. The following publicly available datasets were used in this project: GRCh38 reference genome [https://www.ensembl.org/index.html]. High-throughput sequencing data generated in this study have been deposited in the GEO database under accession code GSE207453. DDX5 RIP-seq data used in this study are available in the GEO database under accession code GSE175455. Source data are provided with this paper.

## Source data

Source Data

## Code availability

All software and tools used for this work are described in the “Methods” section or in the figure legends. Additional dedicated scripts developed for this work are available from the corresponding authors upon request.

## References

[1] Wilusz JE (2018). A 360° view of circular RNAs: from biogenesis to functions. Wiley Interdiscip. Rev. RNA.

[2] Di Timoteo, G., Rossi, F. & Bozzoni, I. Circular RNAs in cell differentiation and development. *Development* **147**, dev182725. (2020). 10.1242/dev.182725 32839270

[3] Nielsen AF (2022). Best practice standards for circular RNA research. Nat. Methods.

[4] Jeck WR (2013). Circular RNAs are abundant, conserved, and associated with ALU repeats. RNA.

[5] Westholm JO (2014). Genome-wide analysis of drosophila circular RNAs reveals their structural and sequence properties and age-dependent neural accumulation. Cell Rep..

[6] Zhang X-O (2014). Complementary sequence-mediated exon circularization. Cell.

[7] Conn SJ (2015). The RNA binding protein quaking regulates formation of circRNAs. Cell.

[8] Ashwal-Fluss R (2014). circRNA biogenesis competes with Pre-mRNA splicing. Mol. Cell.

[9] Errichelli L (2017). FUS affects circular RNA expression in murine embryonic stem cell-derived motor neurons. Nat. Commun..

[10] Knupp, D., Cooper, D. A., Saito, Y., Darnell, R. B. & Miura, P. NOVA2 regulates neural circRNA biogenesis. *Nucleic Acids Res.* **49**, 2849–6862 (2021). 10.1093/nar/gkab523 PMC8266653 34157123

[11] Fei, T. et al. Genome-wide CRISPR screen identifies HNRNPL as a prostate cancer dependency regulating RNA splicing. *Proc. Natl. Acad. Sci.* **114**, E5207-E5215 (2017). 10.1073/pnas.1617467114 PMC5495225 28611215

[12] Kramer MC (2015). Combinatorial control of Drosophila circular RNA expression by intronic repeats, hnRNPs, and SR proteins. Genes Dev..

[13] Liang D (2017). The output of protein-coding genes shifts to circular RNAs when the pre-mRNA processing machinery is limiting. Mol. Cell.

[14] Desrosiers R, Friderici K, Rottman F (1974). Identification of methylated nucleosides in messenger RNA from Novikoff hepatoma cells. Proc. Natl Acad. Sci..

[15] Agarwala SD, Blitzblau HG, Hochwagen A, Fink GR (2012). RNA methylation by the MIS complex regulates a cell fate decision in yeast. PLoS Genet..

[16] Geula S (2015). m 6 A mRNA methylation facilitates resolution of naïve pluripotency toward differentiation. Science.

[17] Zhong S (2008). MTA is an arabidopsis messenger RNA adenosine methylase and interacts with a homolog of a sex-specific splicing factor. Plant Cell.

[18] Zhou C (2017). Genome-wide maps of m6A circRNAs identify widespread and cell-type-specific methylation patterns that are distinct from mRNAs. Cell Rep..

[19] Di Timoteo G (2020). Modulation of circRNA metabolism by m6A modification. Cell Rep..

[20] Dasgupta R, Fuchs J, Rodeberg D (2016). Rhabdomyosarcoma. Semin. Pediatr. Surg..

[21] Ognjanovic S, Linabery AM, Charbonneau B, Ross JA (2009). Trends in childhood rhabdomyosarcoma incidence and survival in the United States, 1975-2005. Cancer.

[22] Schaaf GJ (2005). Full transcriptome analysis of rhabdomyosarcoma, normal, and fetal skeletal muscle: statistical comparison of multiple SAGE libraries. FASEB J..

[23] Sun X (2015). Rhabdomyosarcoma: advances in molecular and cellular biology. Sarcoma.

[24] Skapek SX (2019). Rhabdomyosarcoma. Nat. Rev. Dis. Prim..

[25] Rossi F (2021). CircVAMP3: a circRNA with a role in alveolar rhabdomyosarcoma cell cycle progression. Genes (Basel).

[26] Rossi F (2019). Circ-ZNF609 regulates G1-S progression in rhabdomyosarcoma. Oncogene.

[27] Rossi, F. et al. Circular RNA ZNF609/CKAP5 mRNA interaction regulates microtubule dynamics and tumorigenicity. *Mol. Cell.* **82**, 75–89.e9 (2021). 10.1016/j.molcel.2021.11.032 PMC8751636 34942120

[28] Gao, Y., Wang, J. & Zhao, F. CIRI: An efficient and unbiased algorithm for de novo circular RNA identification. *Genome Biol.* **16**, 4 (2015). 10.1186/s13059-014-0571-3 PMC4316645 25583365

[29] Zhang XO (2016). Diverse alternative back-splicing and alternative splicing landscape of circular RNAs. Genome Res..

[30] Cheng J, Metge F, Dieterich C (2016). Specific identification and quantification of circular RNAs from sequencing data. Bioinformatics.

[31] Liu J (2014). A METTL3–METTL14 complex mediates mammalian nuclear RNA N6-adenosine methylation. Nat. Chem. Biol..

[32] Dardenne E (2014). RNA helicases DDX5 and DDX17 dynamically orchestrate transcription, miRNA, and splicing programs in cell differentiation. Cell Rep..

[33] Legrand JMD (2019). DDX5 plays essential transcriptional and post-transcriptional roles in the maintenance and function of spermatogonia. Nat. Commun..

[34] Caretti G (2006). The RNA helicases p68/p72 and the noncoding RNA SRA are coregulators of MyoD and skeletal muscle differentiation. Dev. Cell.

[35] Gualtieri, A. et al. The RNA helicase DDX5 cooperates with EHMT2 to sustain alveolar rhabdomyosarcoma growth. *Cell. Rep.* **40**, 111267 (2022). 10.1016/j.celrep.2022.111267 36044855

[36] Xu J (2021). The RNA helicase DDX5 promotes viral infection via regulating N6-methyladenosine levels on the DHX58 and NFκB transcripts to dampen antiviral innate immunity. PLoS Pathog..

[37] Zhao, W. et al. RNA helicase DDX5 participates in oxLDL-induced macrophage scavenger receptor 1 expression by suppressing mRNA degradation. *Exp Cell Res.* **366**, 114–120 (2018). 10.1016/j.yexcr.2018.03.003 29522752

[38] Suzuki HI (2009). Modulation of microRNA processing by p53. Nature.

[39] Xiao, W. et al. Nuclear m6A reader YTHDC1 regulates mRNA splicing–supplemental information. *Mol. Cell.* **61**, 925 (2016). 10.1016/j.molcel.2016.01.012 26876937

[40] Sergeeva O, Zatsepin T (2021). RNA helicases as shadow modulators of cell cycle progression. Int J. Mol. Sci..

[41] Vo JN (2019). The landscape of circular RNA in cancer. Cell.

[42] Obayashi, T., Kodate, S., Hibara, H., Kagaya, Y. & Kinoshita, K. COXPRESdb v8: an animal gene coexpression database navigating from a global view to detailed investigations. *Nucleic Acids Res.* (2022). 10.1093/nar/gkac983 PMC9825429 36350658

[43] Goya J (2015). FNTM: a server for predicting functional networks of tissues in mouse. Nucleic Acids Res..

[44] Huang, C., Liang, D., Tatomer, D. C. & Wilusz, J. E. A length-dependent evolutionarily conserved pathway controls nuclear export of circular RNAs. *Genes Dev.* (2018). 10.1101/gad.314856.118 PMC6004072 29773557

[45] Chen R-X (2019). N6-methyladenosine modification of circNSUN2 facilitates cytoplasmic export and stabilizes HMGA2 to promote colorectal liver metastasis. Nat. Commun..

[46] Lane DP, Hoeffler WK (1980). SV40 large T shares an antigenic determinant with a cellular protein of molecular weight 68,000. Nature.

[47] Yan H, Zhang L, Cui X, Zheng S, Li R (2022). Roles and mechanisms of the m6A reader YTHDC1 in biological processes and diseases. Cell Death Discov..

[48] Zhu L, Liu Y, Yang Y, Mao XM, Yin ZD (2019). CircRNA ZNF609 promotes growth and metastasis of nasopharyngeal carcinoma by competing with microRNA-150-5p. Eur. Rev. Med. Pharm. Sci..

[49] Altesha M-A, Ni T, Khan A, Liu K, Zheng X (2019). Circular RNA in cardiovascular disease. J. Cell Physiol..

[50] Lukiw, W. J. Circular RNA (circRNA) in Alzheimer’s disease (AD). *Front. Genet.* **4**, 307 (2013). 10.3389/fgene.2013.00307 PMC3875874 24427167

[51] Wang T (2018). Circular RNAs in metabolic diseases. Adv. Exp. Med. Biol..

[52] Bach D-H, Lee SK, Sood AK (2019). Circular RNAs in cancer. Mol. Ther. Nucleic Acids.

[53] Legnini I (2017). Circ-ZNF609 is a circular RNA that can be translated and functions in myogenesis. Mol. Cell.

[54] Liu Z (2019). Circ-ZNF609 promotes carcinogenesis of gastric cancer cells by inhibiting miRNA-145-5p expression. Eur. Rev. Med. Pharm. Sci..

[55] Wu W (2019). CircZNF609 promotes the proliferation and migration of gastric cancer by sponging mir-483-3p and regulating CDK6. Onco Targets Ther..

[56] Wang S (2018). CircZNF609 promotes breast cancer cell growth, migration, and invasion by elevating p70S6K1 via sponging miR-145-5p. Cancer Manag Res..

[57] Liu Z, Liu F, Wang F, Yang X, Guo W (2021). CircZNF609 promotes cell proliferation, migration, invasion, and glycolysis in nasopharyngeal carcinoma through regulating HRAS via miR-338-3p. Mol. Cell Biochem..

[58] Jin C, Zhao W, Zhang Z, Liu W (2019). Silencing circular RNA circZNF609 restrains growth, migration and invasion by upregulating microRNA-186-5p in prostate cancer. Artif. Cells Nanomed. Biotechnol..

[59] He Y (2020). CircZNF609 enhances hepatocellular carcinoma cell proliferation, metastasis, and stemness by activating the Hedgehog pathway through the regulation of miR-15a-5p/15b-5p and GLI2 expressions. Cell Death Dis..

[60] He L (2019). Functions of N6-methyladenosine and its role in cancer. Mol. Cancer.

[61] Linder B (2015). Single-nucleotide-resolution mapping of m6A and m6Am throughout the transcriptome. Nat. Methods.

[62] Schneider CA, Rasband WS, Eliceiri KW (2012). NIH Image to ImageJ: 25 years of image analysis. Nat. Methods.

[63] Bolger AM, Lohse M, Usadel B (2014). Trimmomatic: a flexible trimmer for Illumina sequence data. Bioinformatics.

[64] Martin M (2011). Cutadapt removes adapter sequences from high-throughput sequencing reads. EMBnet J..

[65] Li, H. Aligning sequence reads, clone sequences and assembly contigs with BWA-MEM. 10.48550/arxiv.1303.3997 (2013).

[66] Ensembl 2018 - PubMed. https://pubmed.ncbi.nlm.nih.gov/29155950/.

[67] Robinson MD, McCarthy DJ, Smyth GK (2010). edgeR: a Bioconductor package for differential expression analysis of digital gene expression data. Bioinformatics.

[68] Dobin A (2013). STAR: ultrafast universal RNA-seq aligner. Bioinformatics.

[69] Anders S, Pyl PT, Huber W (2015). HTSeq—a Python framework to work with high-throughput sequencing data. Bioinformatics.

[70] Quinlan AR, Hall IM (2010). BEDTools: a flexible suite of utilities for comparing genomic features. Bioinformatics.

[71] Lorenz R (2011). ViennaRNA package 2.0. Algorithms Mol. Biol..

[72] Robinson JT (2011). Integrative genomics viewer. Nat. Biotechnol..

