# Supplementary material for: The m6A reader YTHDC1 and the RNA helicase DDX5 control the production of rhabdomyosarcoma-enriched circRNAs
Source: Nat Commun. 2023 Apr 5;14:1898. doi: 10.1038/s41467-023-37578-7 (PMC10076346; doi:10.1038/s41467-023-37578-7)
Supplement: Supplementary file 1 — Supplementary Information [file 41467_2023_37578_MOESM1_ESM.pdf]

Supplementary Information

The m^{6}A reader YTHDC1 and the RNA helicase DDX5 control the production of rhabdomyosarcoma-enriched circRNAs

Dario Dattilo[#,1], Gaia Di Timoteo[#,1], Adriano Setti[#1], Andrea Giuliani[1], Giovanna Peruzzi[2], Manuel Beltran Nebot [1], Alvaro Centrón-Broco[1], Davide Mariani[3], Chiara Mozzetta[4], Irene Bozzoni[*1,2,3]

[1] Department of Biology and Biotechnology Charles Darwin, Sapienza University of Rome, Rome 00185, Italy.
[2] Center for Life Nano- & Neuro-Science@Sapienza, Fondazione Istituto Italiano di Tecnologia (IIT), Rome 00161, Italy.
[3] Center for Human Technologies@Istituto Italiano di Tecnologia (IIT), Genoa 16152, Italy.
[4] Institute of Molecular Biology and Pathology (IBPM), National Research Council (CNR) of Italy.

*e-mail: irene.bozzoni@uniroma1.it
These authors contributed equally: Dattilo Dario, Di Timoteo Gaia, Setti Adriano

Supplementary Figure 1

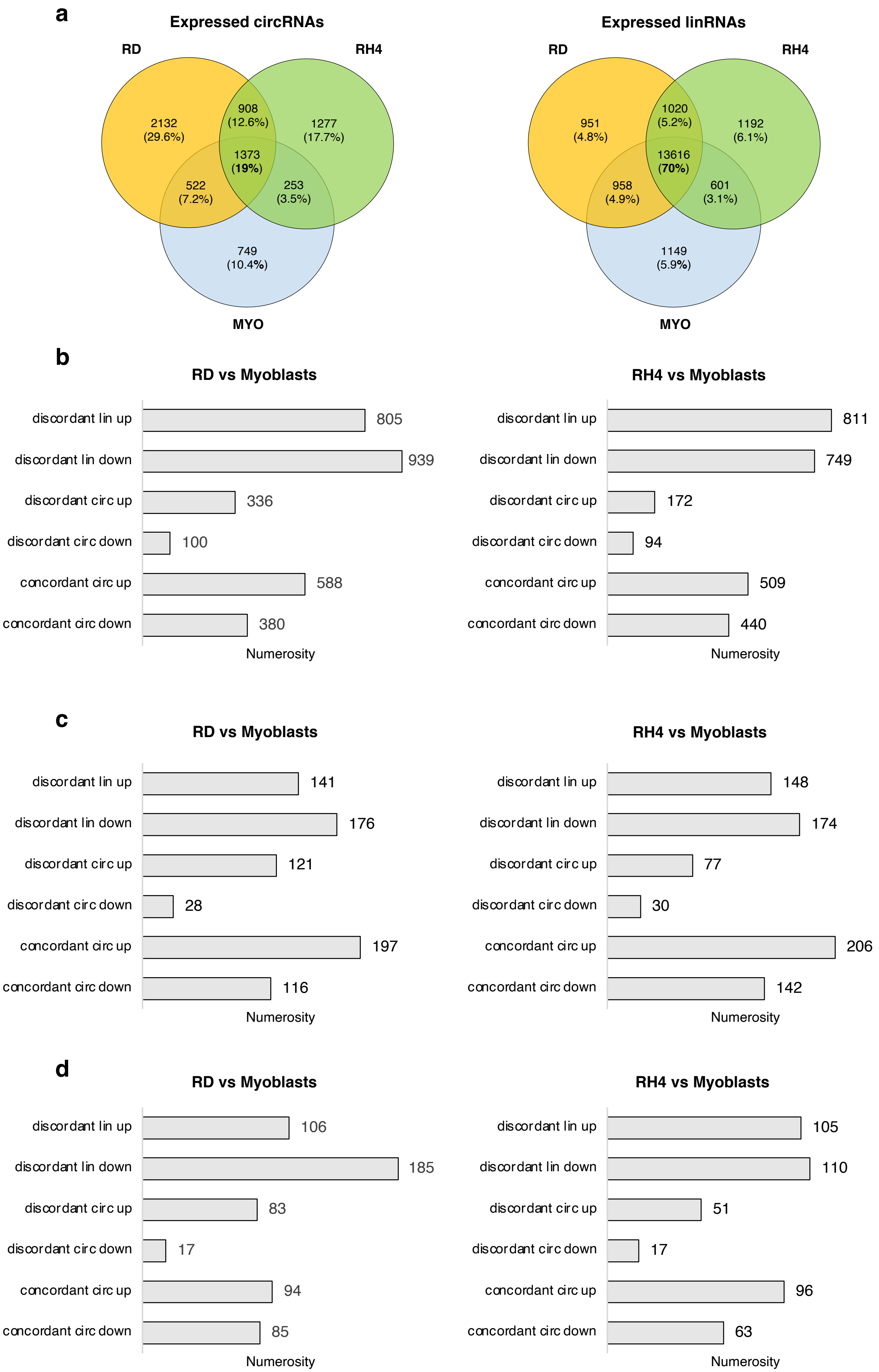

Supplementary Figure 1

e

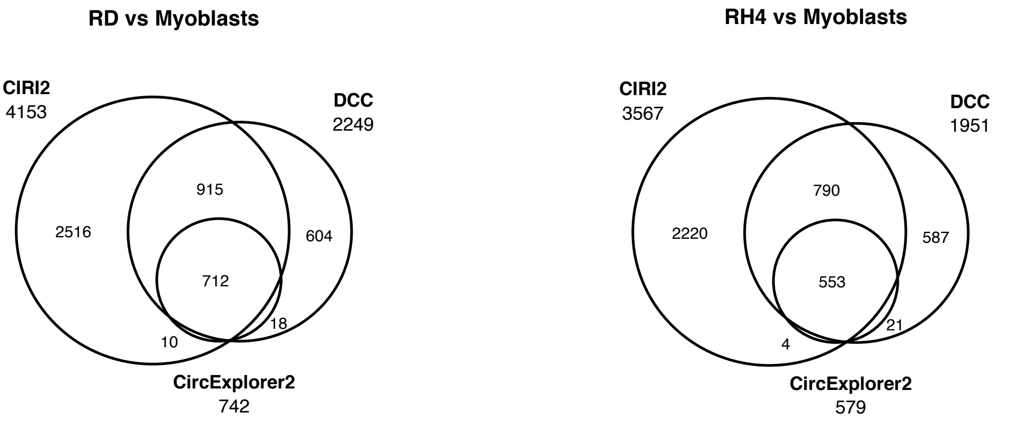

Supplementary Figure 1: CircRNA levels increase in RMS

a, Venn diagrams showing the overlap between circular RNAs (left panel) or linear RNAs (right panel) expressed in wild-type myoblasts (“MYO”), RD cells (“RD”) or RH4 cells (“RH4”). **b, c, d,** Bar-plots depicting the numerosity of different scenarios of deregulation in the comparison between RD and myoblasts (left panel) or between RH4 and myoblasts (right panel), either considering the CIRI2 analysis with all detected circRNAs (B), the top 20% expressed circRNAs (C) or ”*high-confidence*” circRNAs (D).**e,** Venn diagrams showing the overlap between circular RNAs identified by the three tools CIRI2, DCC and CircExplorer2, either in the comparison between RD and myoblasts (left panel) or between RH4 and myoblasts (right panel”). Exact p-values and source data are provided in Source Data file.

Supplementary Figure 2

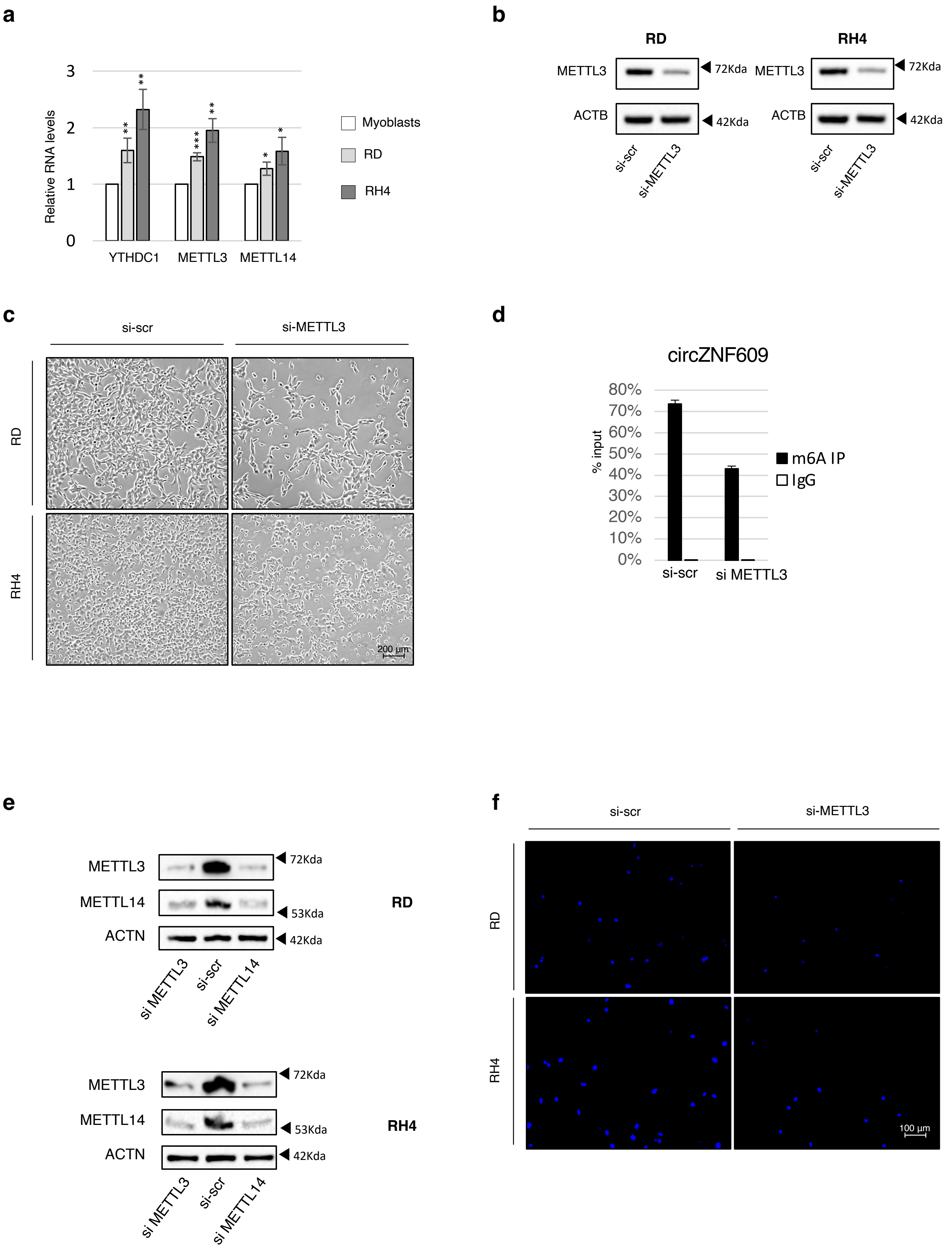

Supplementary Figure 2: m^{6}A factors are altered in RMS and sustain its proliferation and migration rate

a, Relative RNA levels of YTHDC1, METTL3, METTL14 in wild-type myoblasts, RD, and RH4 cell lines. Values are normalized against ACTN1 (RD versus myoblasts) or GAPDH (RH4 versus myoblasts) and expressed as relative quantity with respect to myoblasts set to a value of 1. The relative RNA quantity in the bars is represented as mean of the fold change with standard deviation. The ratio of each sample versus its experimental control was tested by two-tailed Student's t test with correction for multiple test comparison (FDR Benjamini-Hochberg). * indicates a test-derived p-value < 0.05, ** indicate a p-value < 0.01, and *** a p-value < 0.001. n=3 biologically independent replicates. **b,** Representative western blots to evaluate the decrease of METTL3 protein upon its depletion in RD (left panel) or RH4 (right panel) cell lines; ACTB was used as loading control. n=3 biologically independent replicates. **c,** Representative brightfield images of RD (upper panel) or RH4 cells (lower panel) at 48hr transfection with control treatment ("si-scr") or siRNAs against METTL3 ("si-METTL3"). n=4 biologically independent replicates. **d,** Levels of circZNF609 recovered from a representative m^{6}A CLIP in RH4 either in control condition ("si-scr") or upon METTL3 ("si-METTL3"); immunoprecipitation with IgG was used as control. The relative RNA quantity in the bars is represented as mean of technical replicates with standard deviation. n=2 biologically independent replicates. **e,** Representative western blots to evaluate the decrease of METTL3 or METTL14 proteins upon their reciprocal depletion in RD (upper panel) or RH4 (lower panel) cell lines; ACTN was used as loading control. n=3 biologically independent replicates. **f,** Representative images of RD (upper panel) or RH4 cells (lower panel) migrated upon transwell-migration assay after 48hr transfection with control treatment ("si-scr") or siRNAs against METTL3 ("si-METTL3"). Nuclei were stained with DAPI solution. n=4 biologically independent replicates.

Exact p-values and source data are provided in Source Data file.

Supplementary Figure 3

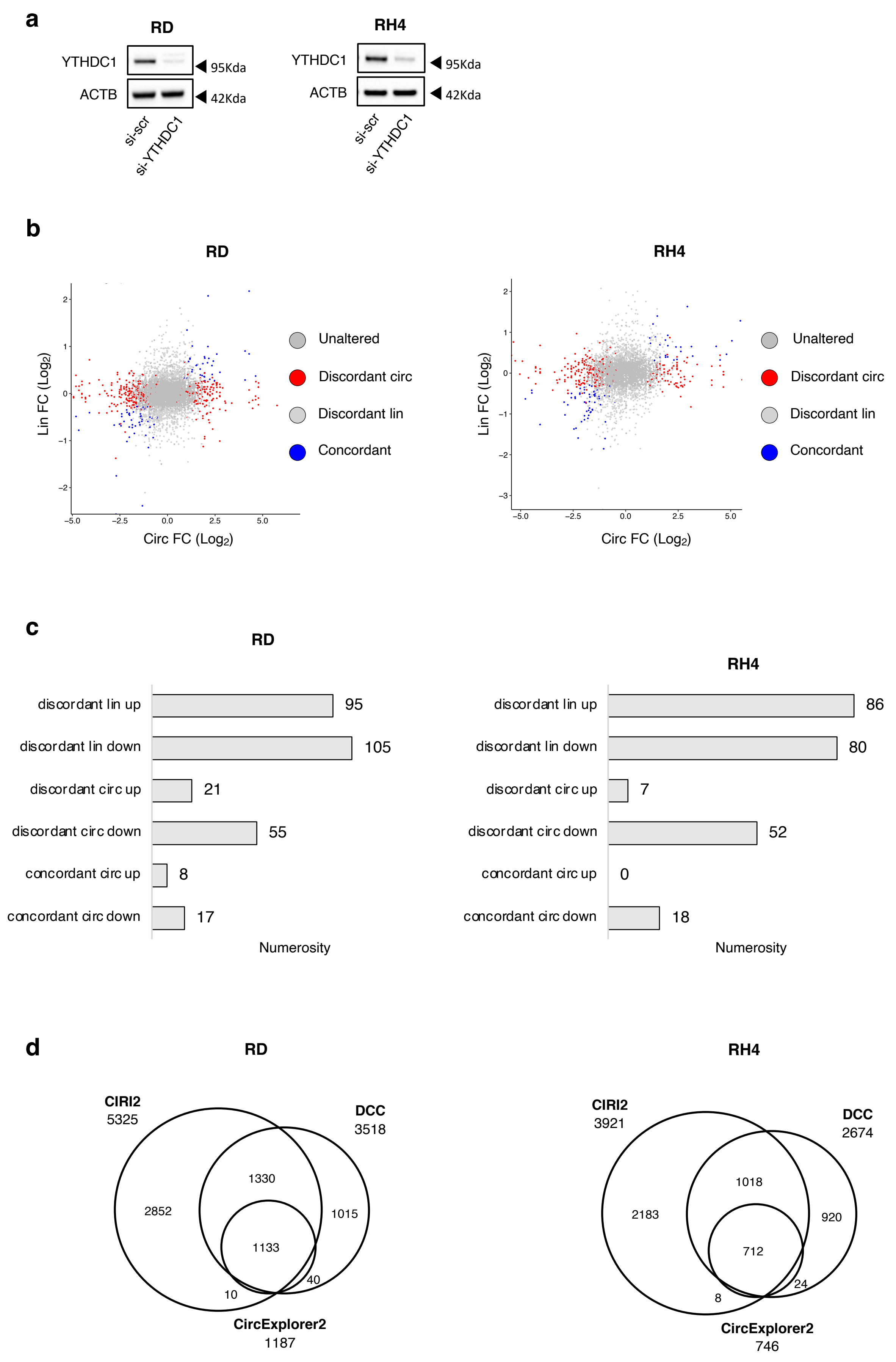

Supplementary Figure 3: YTHDC1 depletion down-regulates circRNAs in RMS

a, Representative western blots to evaluate the decrease of YTHDC1 protein upon its depletion in RD (left panel) or RH4 (right panel) cell lines; ACTB was used as loading control. n=3 biologically independent replicates. **b,** Scatter plots showing, for each circRNA identified in the RNA-seq experiment, the $\log_2$ fold change along with that of its cognate linear RNA upon YTHDC1 depletion in RD (left panel) or in RH4 (right panel). Significantly deregulated circRNAs are indicated by blue dots when their linear counterpart is deregulated in the same direction ("Concordant") and by red dots when the linear is either unaltered or deregulated in the opposite direction ("Discordant circ"); significantly deregulated linRNAs are indicated by light grey dots when their circular counterpart is either unaltered or deregulated in the opposite direction ("Discordant lin"); unaffected circRNAs are indicated by dark grey dots when their linear counterpart is not altered ("Unaltered"). **c,** Bar-plots depicting the numerosity of different scenarios of deregulation in RD (left panel) or RH4 cell lines depleted for YTHDC1, considering "*high-confidence*" circRNAs. **d,** Venn diagrams showing the overlap between circular RNAs identified by the three tools CIRI2, DCC and CircExplorer2 upon YTHDC1 depletion, either in RD (left panel) or in RH4 (right panel) cell lines. Exact p-values and source data are provided in Source Data file.

Supplementary Figure 4

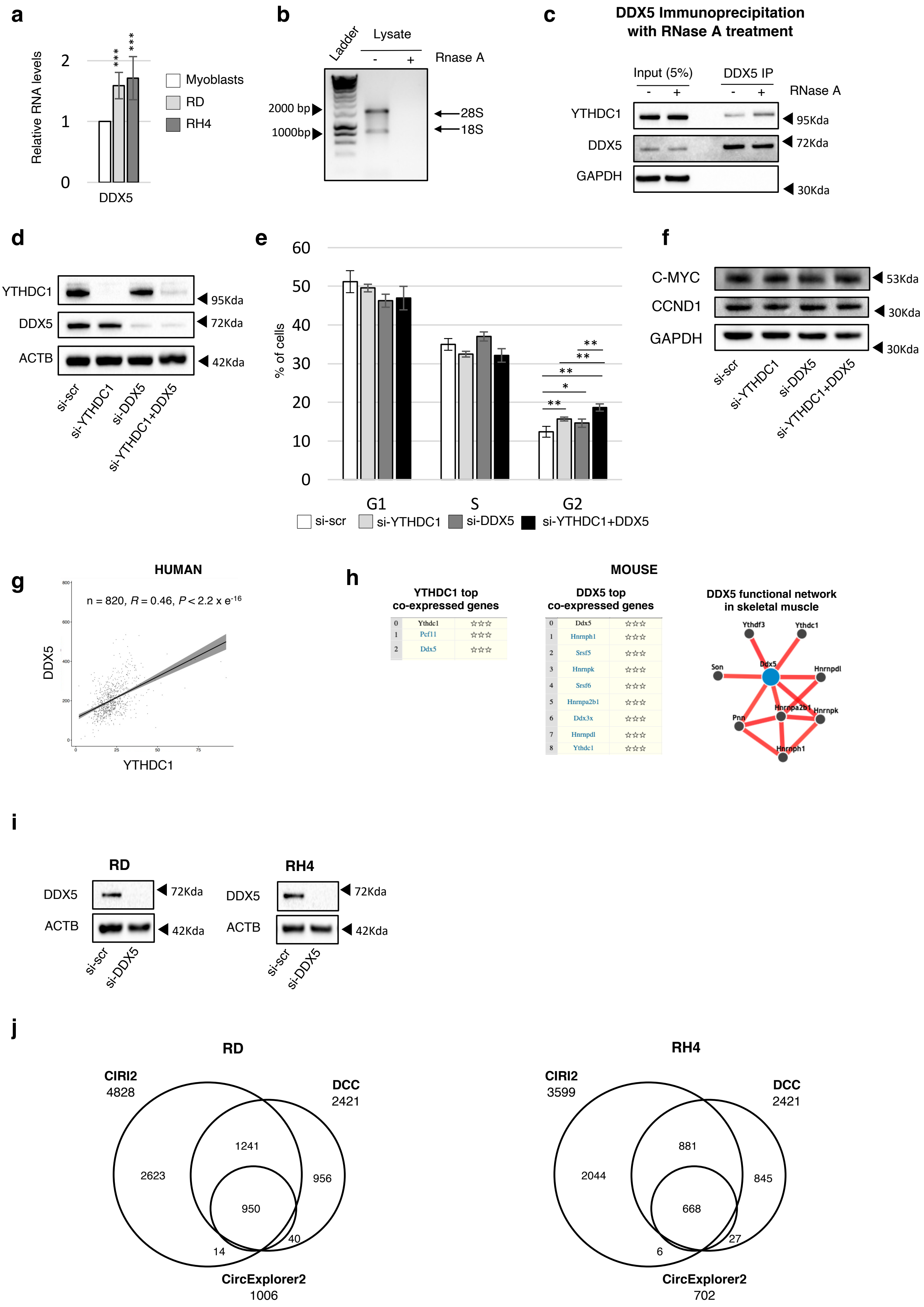

Supplementary Figure 4

k

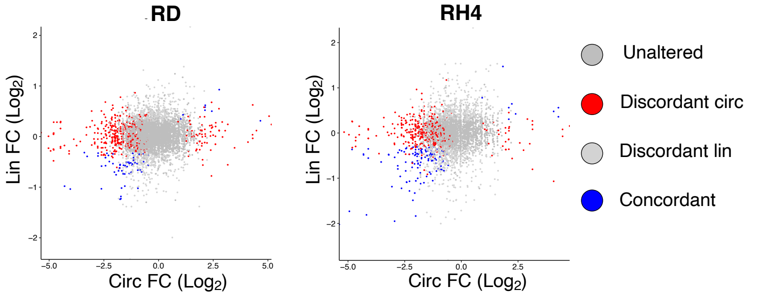

l

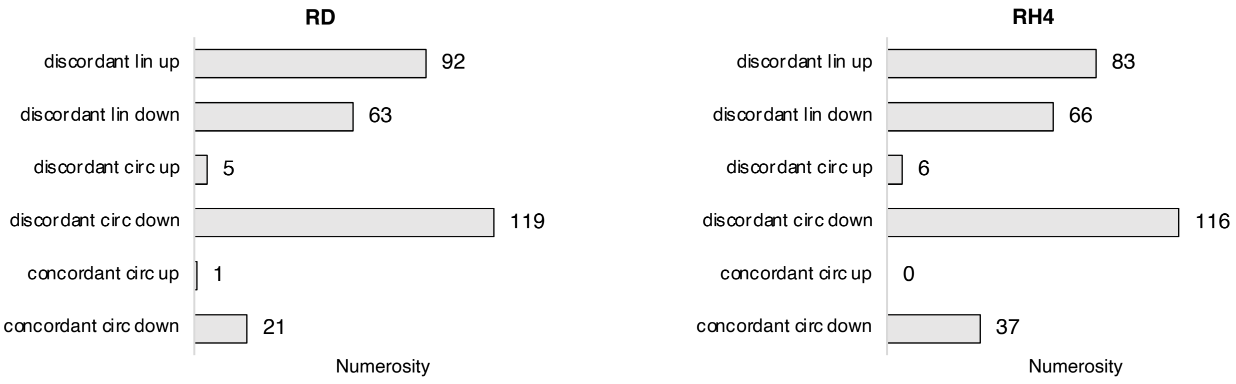

Supplementary Figure 4: DDX5 helicase controls circRNA expression and interacts with YTHDC1

a, Relative RNA levels of DDX5 in wild-type myoblasts, RD, and RH4 cell lines. Values are normalized against ACTN1 (RD versus myoblasts) or GAPDH (RH4 versus myoblasts) and expressed as relative quantity with respect to myoblasts set to a value of 1. The relative RNA quantity in the bars is represented as mean of the fold change with standard deviation. The ratio of each sample versus its experimental control was tested by two-tailed Student's t test with correction for multiple test comparison (FDR Benjamini-Hochberg). * indicates a test-derived p-value < 0.05, ** indicate a p-value < 0.01, and *** a p-value < 0.001. n=3 biologically independent replicates. **b,** Representative agarose gel showing RNase A treatment efficacy on total RNA used as input for the co-IP experiment in Supplementary Fig. 4C. HyperLadder™ 1kb (Meridian Bioscience) was loaded to check the size of rRNA bands. 28S and 18S rRNAs are indicated by arrows. n=3 biologically independent replicates. **c,** Representative western blot analysis of DDX5 immunoprecipitation from whole-cell lysate in RD cells, with or without RNase A treatment. The percentage of input is indicated. GAPDH was used as negative control for the co-immunoprecipitation. n=3 biologically independent replicates. **d,** Representative western blot to evaluate the decrease of YTHDC1 and DDX5 proteins upon their individual or combined depletion in RD cells; ACTB was used as loading control. n=4 biologically independent replicates. **e,** FACS analysis of RD cells upon control treatment ("si-scr"), individual YTHDC1 ("si-YTHDC1") or DDX5 ("si-DDX5") knock-down, or their combined knock-down ("si-YTHDC1+DDX5"). Data are represented as mean percentage of cells in each cell cycle phase ± standard deviation. The ratio of each sample versus its experimental control was tested by two-tailed Student's t test with correction for multiple test comparison (FDR Benjamini-Hochberg). * indicates a test-derived p-value < 0.05, ** indicate a p-value < 0.01, and *** a p-value < 0.001. n=4 biologically independent replicates. **f,** Representative western blot showing the effect of individual or combined depletion of YTHDC1 and DDX5 on selected DDX5 target genes in RD cells; GAPDH was used as loading control. n=4 biologically independent replicates. **g,** Scatter plots depicting the two-sided Pearson correlation between expression (FPKM) of DDX5 and YTHDC1. Dots represent expression counts of each gene retrieved from tumor samples deposited in the MiOncoCirc database (https://mioncocirc.github.io/). **h,** Graphical output from two different databases querying gene expression data in mouse. List of top co-expressed genes of YTHDC1 (left panel) or DDX5 (middle panel) are reported; data are obtained from the COXPRESdb (https://coxpresdb.jp/). Representation of DDX5 most functionally connected interactor genes in skeletal muscle tissue (right panel), according to the FNTM database (http://fntm.princeton.edu/). **i,** Representative western blots to evaluate the decrease of DDX5 protein upon its depletion in RD (left panel) or RH4 (right panel) cell lines; ACTB was used as loading control. n=3 biologically independent replicates. **j,** Venn diagrams showing the overlap between circular RNAs identified by the three tools CIRI2, DCC and CircExplorer2 upon DDX5 depletion, either in RD (left panel) or in RH4 (right panel) cell lines. **k,** Scatter plots showing, for each circRNA identified in the RNA-seq experiment the $\log_2$ fold change along with that of its cognate linear RNA upon DDX5 depletion in RD (left panel) or in RH4 (right panel). Significantly deregulated circRNAs are indicated by blue dots when their linear counterpart is deregulated in the same direction ("Concordant") and by red dots when the linear is either unaltered or deregulated in the opposite direction ("Discordant circ"); significantly deregulated linRNAs are indicated by light grey dots when their circular counterpart is either unaltered or deregulated in the opposite direction ("Discordant lin"); unaffected circRNAs are indicated by dark grey dots when their linear counterpart is not altered ("Unaltered"). **l,** Bar-plots depicting the numerosity of different scenarios of deregulation in RD (left panel) or RH4 cell lines depleted for DDX5, considering *"high-confidence"* circRNAs. Exact p-values and source data are provided in Source Data file.

Supplementary Figure 5

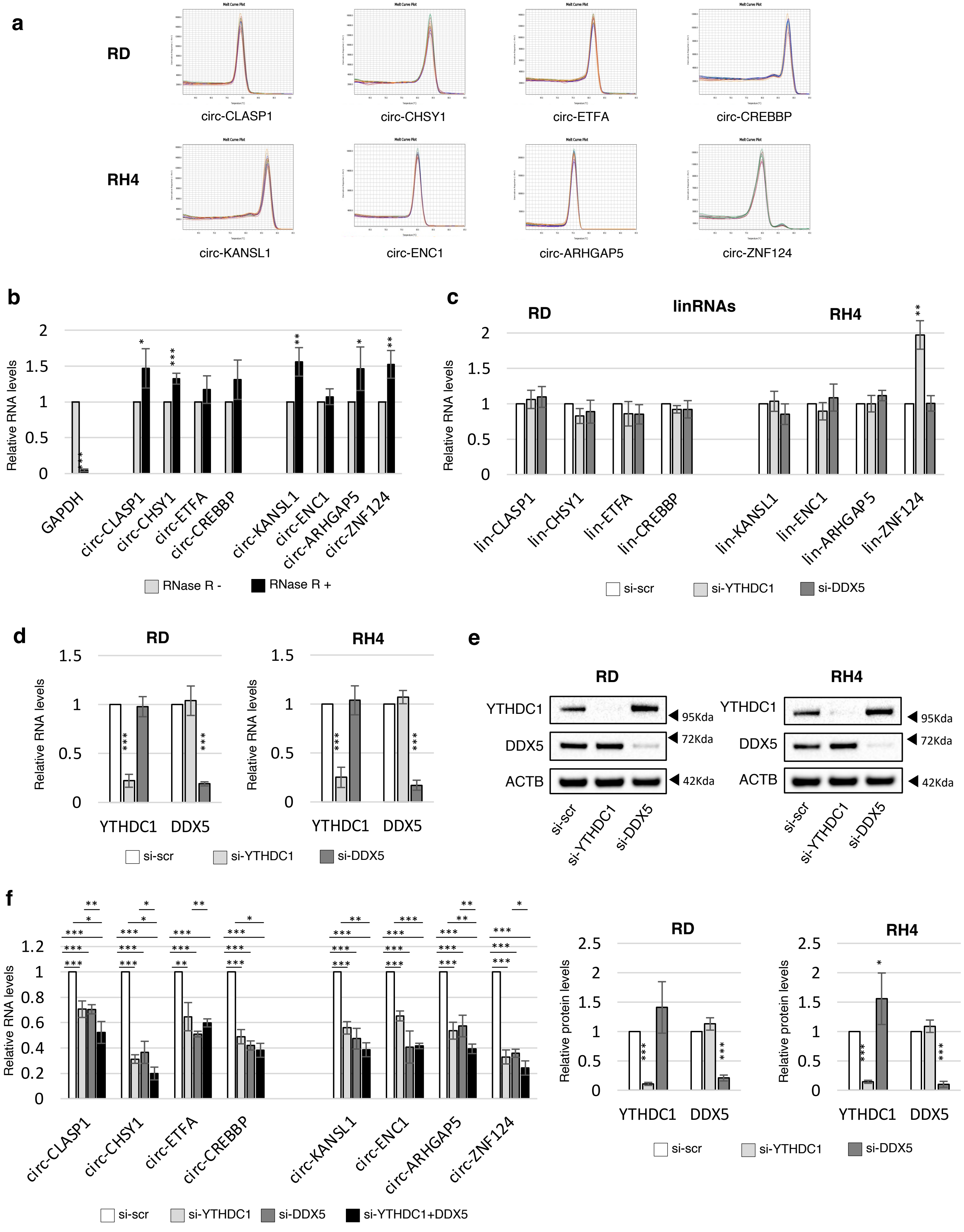

Supplementary Figure 5

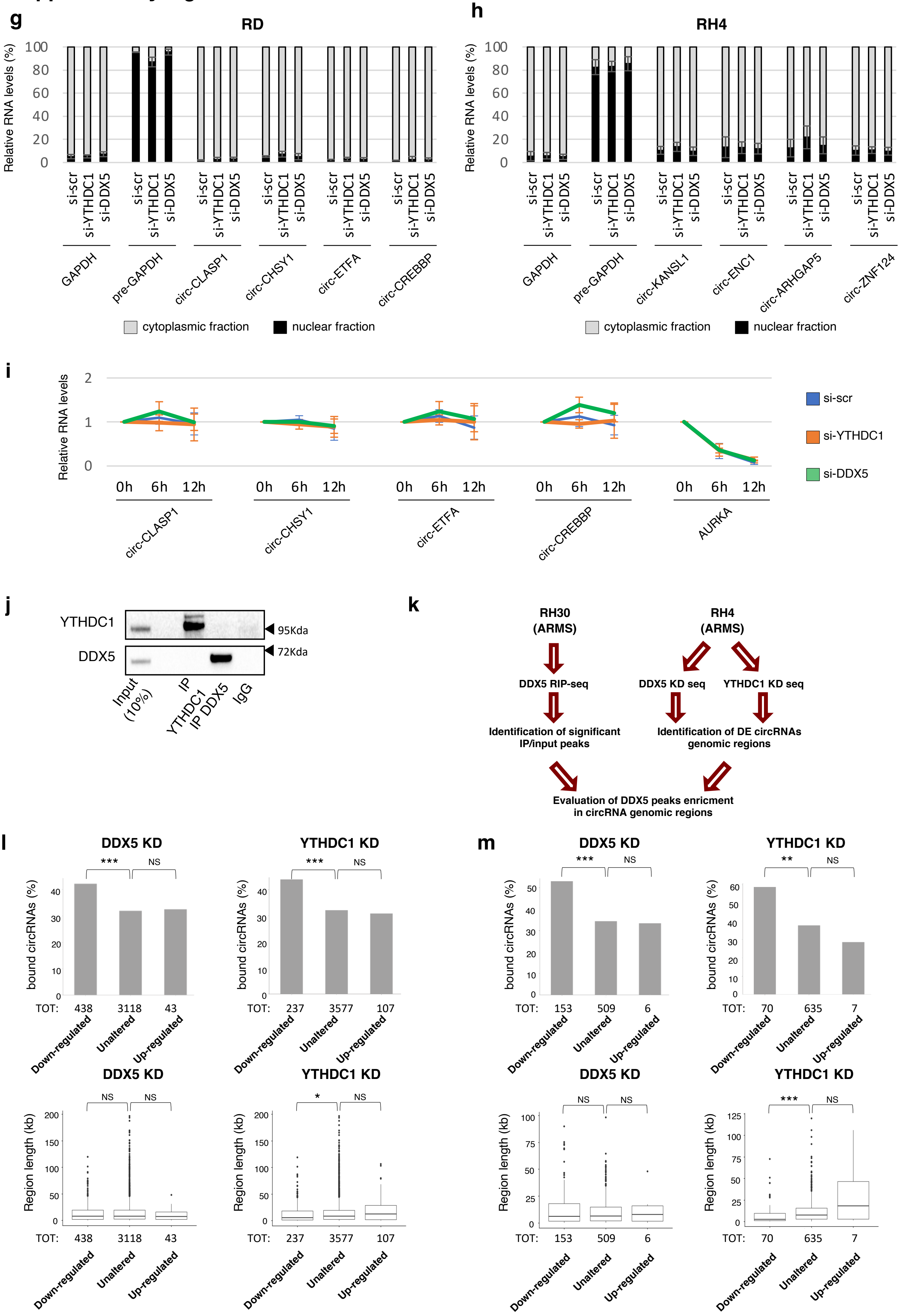

Supplementary Figure 5

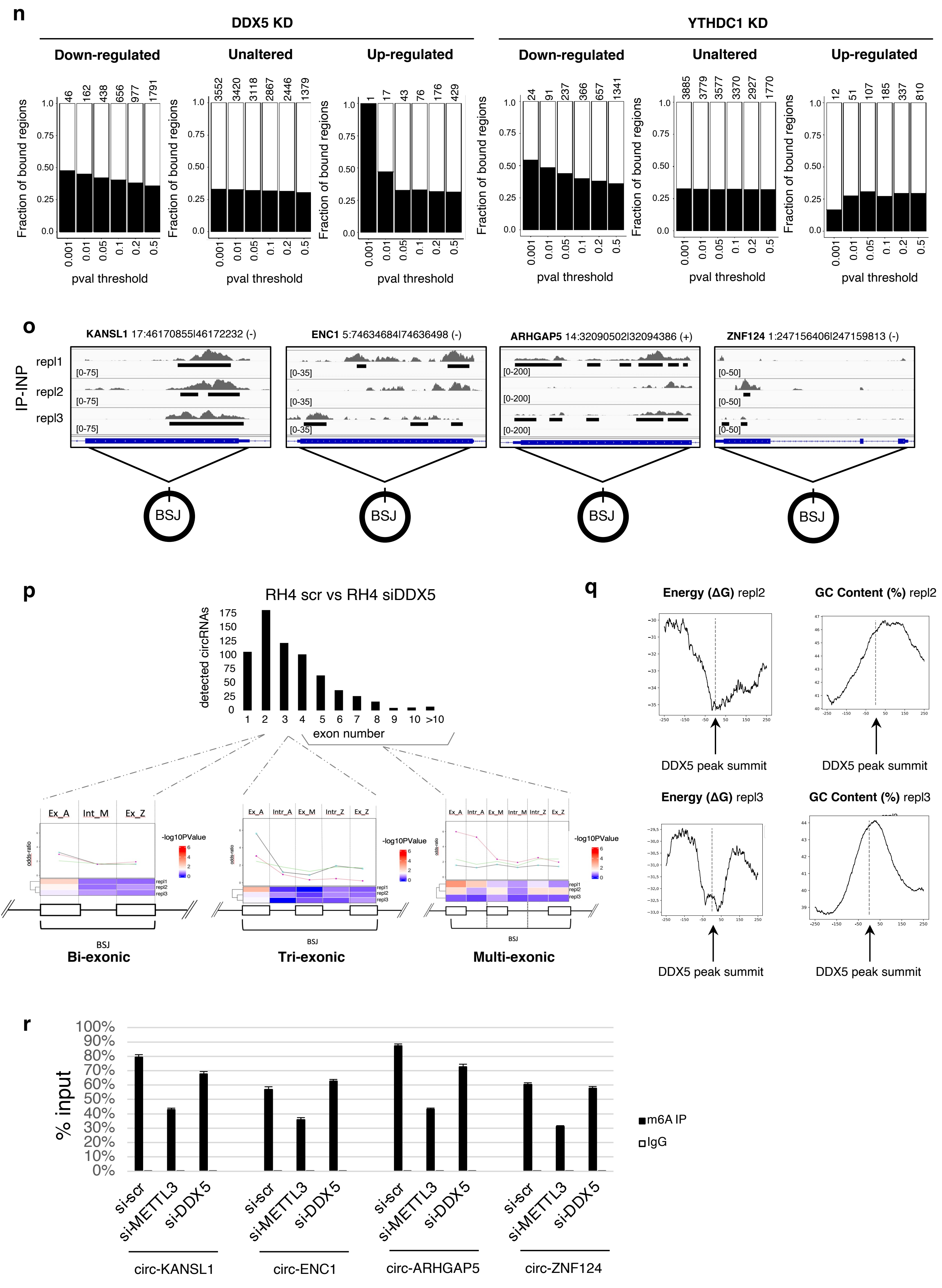

Supplementary Figure 5: DDX5 and YTHDC1 directly regulate a subset of circRNAs promoting their up-regulation in RMS

a, Melt curve plots from the RT-qPCR amplification of selected circRNAs shown in Fig. 5b. **b,** Relative RNA levels of selected circRNAs upon RNase R treatment ("RNase R +") in RD cells. GAPDH was used as positive control for RNase R efficiency. Values are expressed as relative quantity with respect to the untreated condition ("RNase R –") set to a value of 1. The relative RNA quantity in the bars is represented as mean of the fold change with standard deviation. n=4 biologically independent replicates. **c,** Relative RNA levels of selected linear RNAs upon YTHDC1 knock-down ("si-YTHDC1") or DDX5 knock-down ("si-DDX5)" in RD or RH4 cell lines. Values are normalized against GAPDH and expressed as relative quantity with respect to scramble siRNA ("si-scr") treatment set to a value of 1. The relative RNA quantity in the bars is represented as mean of the fold change with standard deviation. n=3 biologically independent replicates. **d,** Relative RNA levels of YTHDC1 and DDX5 transcripts upon their reciprocal depletion in RD cells (left panel) or RH4 cells (right panel). Values are normalized against GAPDH and expressed as relative quantity with respect to scramble siRNA ("si-scr") treatment set to a value of 1. The relative RNA quantity in the bars is represented as mean of the fold change with standard deviation n=4 biologically independent replicates. **e,** Representative western blots to evaluate the decrease of YTHDC1 or DDX5 proteins upon their reciprocal depletion in RD (left panel) or RH4 (right panel) cell lines; ACTB was used as loading control. Quantification of the western blots are reported below. Values were normalized to ACTB and expressed as relative quantity with respect to scramble siRNA ("si-scr") treatment set to a value of 1. The relative protein quantity in the bars is represented as mean of the fold change with standard deviation. n=4 biologically independent replicates. **f,** Relative RNA levels of selected circRNAs upon control treatment ("si-scr"), individual YTHDC1 ("si-YTHDC1") or DDX5 ("si-DDX5") knock-down, or their combined knock-down ("si-YTHDC1+DDX5") in RD or RH4 cell lines. Values are normalized against GAPDH and expressed as relative quantity with respect to scramble siRNA ("si-sc") treatment set to a value of 1. The relative RNA quantity in the bars is represented as mean of the fold change with standard deviation. n=4 biologically independent replicates. **g, h,** Bar-plots showing the percentage in the nuclear and the cytoplasmic fraction in RD (g) or RH4 (h) cell lines for selected circRNAs in control condition ("si-scr") or upon YTHDC1 ("si-YTHDC1") or DDX5 knock-down ("si-DDX5"). GAPDH and pre-GAPDH were used as controls for cell fractionation. The relative RNA quantity in the bars is represented as mean of technical replicates with standard deviation n=3 biologically independent replicates. **i,** Relative RNA levels of selected circRNAs after Actinomycin D treatment in RD cell lines at dfferent time points (0h, 6h, 12h), either in control condition ("si-scr") or upon YTHDC1 ("si-YTHDC1") or DDX5 knock-down ("si-DDX5"). For each condition, values are expressed as relative quantity with respect to 0h time point set to a value of 1. The relative RNA quantity in the bars is represented as mean of the fold change with standard deviation. n=3 biologically independent replicates. **j,** Representative western blot showing YTHDC1 or DDX5 IP efficiency. The percentage of input is indicated. Immunoprecipitation with IgG was used as control. n=3 biologically independent replicates. **k,** Scheme depicting bioinformatic workflow for the analysis of DDX5 binding to circRNAs deregulated upon YTHDC1 and DDX5 knock-down in RH4 cells. **l, m,** Bar plots representing the fraction of all detected circRNAs (l) or "*high-confidence*" circRNAs (m) down-regulated, unaltered or up-regulated upon either DDX5 (left panel) or YTHDC1 knock-down (right panel) in RH4 identified as DDX5 binding targets. P-values for differences between proportions were calculated using two-sided Fisher exact test. *** indicates a p-value < 0.001. Box plots depicting the length of genomic regions involved in circularization for each circRNA category analyzed are reported below. For genomic lengths, the differences between groups were calculated using Mann-Whitney U test. Numbers of each group – representing the sum of bound and unbound targets - are reported below both the bars and the boxes. See methods section for statistical analyses details. **n,** Bar plots representing the fraction of circRNAs down-regulated, unaltered or up-regulated upon either DDX5 (left panel) or YTHDC1 knock-down (right panel) and identified as DDX5 targets using different p-value cutoffs to select differentially expressed circRNAs in knock-down condition. Numbers of each group – representing the sum of bound and unbound targets - are reported above the bars. See methods section for statistical analyses details. **o**, IGV screens depicting examples of DDX5 RIP-seq peaks on validated circRNAs in RH4 cell line. Coverage (CPM) of IP-INP signal for each replicate of the DDX5 RIP-seq is represented, peak regions are depicted as black lines, transcriptome annotation is depicted with a graphic representation of regions involved in circularization. **p,** Figure representing the DDX5 binding enrichment analysis for each RIP replicate on on bi-exonic, three-exonic and multi-exonic circRNAs (*high-confidence*), comparing down-regulated circRNAs upon DDX5 depletion (RH4 cell line) to invariant circRNAs. The bar plot on the top depicts *high-confidence* circRNAs counts for each circRNA type with the specified exon number. Heatmaps on the bottom depict, for each circRNA category, $-\log_{10}$ p-value associated to each region. The line plots annotation represents the associated odds-ratio. Statistical significance was assessed using two-sided Fisher's exact test. **q**, Line plots representing ΔG (left panel) and GC content (right panel) of 500nt regions centered to DDX5 peak summit of RIP-Seq replicates 2 and 3 .

r, Levels of selected circRNAs recovered from a representative m^6A CLIP in RH4 either in control condition (“si-scr”) or upon METTL3 (“si-METTL3”) or DDX5 knock-down (“si-DDX5”); immunoprecipitation with IgG was used as control. The relative RNA quantity in the bars is represented as mean of technical replicates with standard deviation. n=2 biologically independent replicates. In all panels, the ratio of each sample versus its experimental control was tested by two-tailed Student’s t test, either without correction (b) or with correction for multiple test comparison (FDR Benjamini-Hochberg) (c,d,e,f,g,h,i). * indicates a test-derived p-value < 0.05, ** indicate a p-value < 0.01, and *** a p-value < 0.001. Exact p-values and source data are provided in Source Data file.

Supplementary Table Legends

Supplementary Table 1

DNA oligonucleotides used in this study.

Oligonucleotide name	Sequence (5'-3')	Supplier
ACTN1_fw	ATCAACTTCAACACGCTGCA	Bio-Fab Research
ACTN1_rev	AGCCTCCGGATCTCATTCAG	Bio-Fab Research
GAPDH_fw	CACCATCTTCCAGGAGTGAG	Bio-Fab Research
GAPDH_rev	CCTTCTCCATGGTGGTGAAGAC	Bio-Fab Research
pre-GAPDH_fw	CTGGGGGTAAGGAGATGCTG	Bio-Fab Research
pre-GAPDH_rev	TTACCAGAGTTAAAAGCAGCCC	Bio-Fab Research
YTHDC1_fw	CCTACGCCAGATGGTTCTGAG	Bio-Fab Research
YTHDC1_rev	CATTCTCAGTGTTGTTCCCTTGC	Bio-Fab Research
METTL3_fw	AAGCAGCTGGACTCTCTGCG	Bio-Fab Research
METTL3_rev	GCACTGGGCTGTCACTACGG	Bio-Fab Research
METTL14_fw	GCAGTTGGGAGCTGAAAGTG	Bio-Fab Research
METTL14_rev	GGAAGCCCTGCAAGTTTCTC	Bio-Fab Research
DDX5_fw	GCCGGGACCGAGGGTTTGGT	Bio-Fab Research
DDX5_rev	CTTGTGCTGTGCGCCTAGCCA	Bio-Fab Research
ZNF609_fw_circ	AACCGGAGCCAGAGGAAGG	Bio-Fab Research
ZNF609_rev_circ	AGCTATGTTCTCAGACCTGC	Bio-Fab Research
CLASP1_fw	TGGATGGACTTGCTACCTCTT	Bio-Fab Research
CLASP1_rev_circ	TAAAGGAGTGAGTGGCAGAGT	Bio-Fab Research
CLASP1_rev_lin	CTGCTCCCTCACAGAGTCTT	Bio-Fab Research
CLASP1_rev_pre	CTAGAAACCAAGGAAGCATGC	Bio-Fab Research
CHSY1_fw	AGTGTCTCCGGGAGATGTAC	Bio-Fab Research
CHSY1_rev_circ	TACAGATGTGTCAGAACCCTCA	Bio-Fab Research
CHSY1_rev_lin	GGGTGTAATGTGATAGCTTGGT	Bio-Fab Research
CHSY1_rev_pre	GTTTCCTATGATACGCTCAGAAC	Bio-Fab Research
ETFA_fw	TGCTGCTGTTGATGCTGGCT	Bio-Fab Research
ETFA_rev_circ	AGTAATGGGTGCTAGGGAATC	Bio-Fab Research
ETFA_rev_lin	GCAACTATTCCATAATCTGCCAC	Bio-Fab Research
ETFA_rev_pre	GCTAACTGCTCAGGAACACTG	Bio-Fab Research
CREBBP_fw	AGCTGGAATGCCGTACCCTA	Bio-Fab Research
CREBBP_rev_circ	ATTGGGTATCAGCTCATCAGGA	Bio-Fab Research
CREBBP_rev_lin	CTTGACTAAAGGGCTGTCCAA	Bio-Fab Research
CREBBP_rev_pre	CCGCGGTTAGGTAGGAAGTA	Bio-Fab Research
KANSL1_fw	TGAAGAGGAAGAACTGACCAG	Bio-Fab Research
KANSL1_rev_circ	ATTCAGCACAGAGAGACAGGA	Bio-Fab Research
KANSL1_rev_lin	GTCTGTTTGCTGACGAATTCG	Bio-Fab Research
ENC1_fw	TACTGCATTTGTCAGCACCTG	Bio-Fab Research
ENC1_rev_circ	TGGACTTGCGGTTCTCATGC	Bio-Fab Research
ENC1_rev_lin	CCTTCATGTCCAGTTAGAGCAT	Bio-Fab Research
ARHGAP5_fw	GTGAAAGAAGATAAAAAGGAAGATG	Bio-Fab Research
ARHGAP5_rev_circ	AAGGTAACCAGATATGCTTCTC	Bio-Fab Research
ARHGAP5_rev_lin	GAGGGGCATCCCAAAGTAAT	Bio-Fab Research
ZNF124_fw_circ	ACTGTGAATGTAAGGTGTAGGAA	Bio-Fab Research
ZNF124_fw_lin	CTTCACTCTCGGCGGTTCAG	Bio-Fab Research
ZNF124_rev	TCCAACAAAGCCCACTCCT	Bio-Fab Research
VAMP3_fw_circ	ATGTGGGCAATCGGGATTAC	Bio-Fab Research
VAMP3_rev_circ	GCACGGTCGTCTAACTCAGA	Bio-Fab Research
G9A_fw	AGAGTGTGGACGGAGAGCTC	Bio-Fab Research
G9A_rev	GGTCTCCCGCTTGAGGAT	Bio-Fab Research
RNA spike-In_fw	TTCTCTCTGTAGCCCCGACTGT	Bio-Fab Research
RNA spike-In_rev	CTCTCCCCTTCTTCACTTTCTTTC	Bio-Fab Research
AURKA_fw	TTCCTCCGTCCCTGAGTGT	Bio-Fab Research
AURKA_rev	GGTCCATGATGCCTCTAGC	Bio-Fab Research
DNA spike_T7_fw	TAATACGACTCACTATAGGG	Bio-Fab Research
DNA spike_BGH_rev	GCCTGGCAACTAGAAGGCA	Bio-Fab Research

Supplementary Table 2
SiRNAs used in this study.

siRNA name	Sense Sequence	Supplier	Catalog No.
Si-scramble (Ctrl)	AAT TCT CCG AAC GTG TCA CGT	Qiagen	1022076
Si-METTL3_1	AGG AGC CAG CCA AGA AAT CAA	Qiagen	SI04340749
Si-METTL3_2	CTG CAA GTA TGT TCA CTA TGA	Qiagen	SI04317096
Si-METTL3_3	CAG GAG ATC CTA GAG CTA TTA	Qiagen	SI04241265
Si-METTL3_4	CCG CGT GAG AAT TGG CTA TAT	Qiagen	SI04140038
Si-METTL14	CTGATAGAGTTCAGCTAATTA	Qiagen	SI05069463
Si-YTHDC1	CCA GAA GAT TAT GAT ATT CAT	Qiagen	SI00764680
Si-DDX5	AAC CGC AAC CAU UGA CGC CAU	Sigma-Aldrich	Custom

Supplementary Table 3
Antibodies used in this study.

Antibody name	Supplier	Catalog No.	Dilution
Anti-m6A polyclonal antibody	Abcam	ab151230	1:100
Anti-METTL3 [EPR18810] monoclonal antibody	Abcam	ab195352	1:1000
Anti-METTL14 polyclonal antibody	Atlas	HPA038002	1:1000
Anti-ACTB-Peroxidase (AC-15) monoclonal antibody	Sigma-Aldrich	A3854	1:2500
Anti-YTHDC1 polyclonal antibody	Abcam	ab122340	1:500
Anti-DDX5 polyclonal antibody	Cell Signalling	9877	1:1000
Anti-DDX5 polyclonal antibody	Abcam	ab10261	1:100
Anti-DROSHA polyclonal antibody	Abcam	ab12286	1:1000
Anti-SRSF3 polyclonal antibody	Abcam	ab125124	1:500
Anti-GAPDH (6C5) monoclonal antibody	Santa Cruz Biotechnology	sc-32233	1:1000
Anti-CCND1 (72-13G) monoclonal antibody	Santa Cruz Biotechnology	sc-450	1:500
Anti-c-MYC (9E10) monoclonal antibody	Santa Cruz Biotechnology	sc-40	1:500
Anti-ACTN (H-300) polyclonal antibody	Santa Cruz Biotechnology	sc-15335	1:1000
Anti-Rabbit IgG (H+L) Secondary Antibody, HRP	Thermo Fisher Scientific	31460	1:10000
Anti-Mouse IgG (H+L) Secondary Antibody, HRP	Thermo Fisher Scientific	32430	1:10000
Anti-Goat IgG (H+L) Secondary Antibody, HRP	Thermo Fisher Scientific	31402	1:10000
