# Supplementary material for: The m6A reader YTHDC1 and the RNA helicase DDX5 control the production of rhabdomyosarcoma-enriched circRNAs
Source: Nat Commun. 2023 Apr 5;14:1898. doi: 10.1038/s41467-023-37578-7 (PMC10076346; doi:10.1038/s41467-023-37578-7)
Supplement: Supplementary file 2 — Description of Additional Supplementary Files [file 41467_2023_37578_MOESM2_ESM.pdf]

Description of Additional Supplementary Files

File Name: Supplementary Data 1
Description: **Results of the differential expression analyses.** Differential expression analyses of circular and linear splicing events between specified conditions. In each panel, the following columns are reported: coordinates of the splicing event ("Position"); typology of the splicing event ("type"), either circular or linear; ensembl stable gene ID ("Gene_ID") and gene symbol ("Gene_Name") of circRNA host gene; mean of normalized CPMs for samples belonging to the first condition and to the second condition; base 2 logarithm of fold change between conditions ("logFC"); logarithm of counts per million reads ("logCPM"); log-likelihood ratio test metric ("LR"); p value indicating the significance of splicing event deregulation ("Pvalue"); adjusted p-value with Benjamini-Hochberg multi-test correction ("FDR"); programs that are able to detect the circRNA in the related conditions ("Detected_By"); circAtlas ID related to the circRNA, when available otherwise "none" ("circAtlas_ID"). See methods section for statistical analyses details.
