# Supplementary material for: The m6A reader YTHDC1 and the RNA helicase DDX5 control the production of rhabdomyosarcoma-enriched circRNAs
Source: Nat Commun. 2023 Apr 5;14:1898. doi: 10.1038/s41467-023-37578-7 (PMC10076346; doi:10.1038/s41467-023-37578-7)
Supplement: Supplementary file 4 — Reporting Summary [file 41467_2023_37578_MOESM4_ESM.pdf]

nature portfolio

Corresponding author(s): Irene Bozzoni

Last updated by author(s): 16/03/2023

Reporting Summary

Nature Portfolio wishes to improve the reproducibility of the work that we publish. This form provides structure for consistency and transparency in reporting. For further information on Nature Portfolio policies, see our Editorial Policies and the Editorial Policy Checklist.

Statistics

For all statistical analyses, confirm that the following items are present in the figure legend, table legend, main text, or Methods section.

n/a	Confirmed	
☐	☒	The exact sample size (*n*) for each experimental group/condition, given as a discrete number and unit of measurement
☐	☒	A statement on whether measurements were taken from distinct samples or whether the same sample was measured repeatedly
☐	☒	The statistical test(s) used AND whether they are one- or two-sided<br>*Only common tests should be described solely by name; describe more complex techniques in the Methods section.*
☒	☐	A description of all covariates tested
☐	☒	A description of any assumptions or corrections, such as tests of normality and adjustment for multiple comparisons
☐	☒	A full description of the statistical parameters including central tendency (e.g. means) or other basic estimates (e.g. regression coefficient) AND variation (e.g. standard deviation) or associated estimates of uncertainty (e.g. confidence intervals)
☐	☒	For null hypothesis testing, the test statistic (e.g. *F*, *t*, *r*) with confidence intervals, effect sizes, degrees of freedom and *P* value noted<br>*Give P values as exact values whenever suitable.*
☒	☐	For Bayesian analysis, information on the choice of priors and Markov chain Monte Carlo settings
☒	☐	For hierarchical and complex designs, identification of the appropriate level for tests and full reporting of outcomes
☐	☒	Estimates of effect sizes (e.g. Cohen's *d*, Pearson's *r*), indicating how they were calculated

Our web collection on statistics for biologists contains articles on many of the points above.

Software and code

Policy information about availability of computer code

Data collection	Agarose gels and western blots were acquired thanks to a ChemiDocTM MP Imager (Bio-Rad). DNA amplification in RT-qPCR was monitored with an Applied BiosystemsTM 7S00 Fast or StepOnePlus System qPCR instrument. The samples for flow cytometry analysis of cell cycle were processed using a BD LSRFortessa (BD Biosciences, San Jose, CA, USA). Images for transwell-migration assay were acquired using an inverted microscope Zeiss Axio Observer A1 Phase Contrast supported with Zeiss AxioCam MRm camera. Images were acquired using a Zeiss Plan-Neofluar 10x objective (NA 0.3) and were collected with the AuxioVision software (Zeiss) version 4.8.2. All RNA-seq samples were sequenced on an IIlumina Novaseq 6000 Sequencing system (IIlumina, San Diego, CA).
Data analysis	Image LabTM 5.2.1 Software (BioRad) was used for the acquisition and analysis of agarose gels and western blots. DNA amplification in RT-qPCR was analyzed with the 7500 Software (Applied Biosystems) version 2.3 or with the StepOneTM Software (Applied Biosystems) version 2.3. Flow cytometry analysis were carried out using the FlowJoTM computer software (TreeStar, Ashland, OR, USA) version 9.3.2. Images for transwell-migration assay were analyzed using the the AuxioVision software (Zeiss) version 4.8.2. For bioinformatic analyses several tools were used including Trimmomatic (v0.39), Cutadapt (v3.2), Bowtie2 (v2.4.2), BWA-MEM (v0.7.17), CIRI2 (v2.0.6), circExplorer2 (v2.3.8), DCC (v0.5.0), edgeR (v3.34.1), HTSeq (v0.13.5), STAR (v2.7.7a), Picard suite (v2.24.1), SAMtools (v1.10), bedtools suite (v2.29.1), RNAfold (v2.4.17), deepTools (v3.5.1), ComplexHeatmap (v2.8.0) and IGV (v2.11.9). When needed data were further processed with Microsoft Excel version 16.71.

For manuscripts utilizing custom algorithms or software that are central to the research but not yet described in published literature, software must be made available to editors and reviewers. We strongly encourage code deposition in a community repository (e.g. GitHub). See the Nature Portfolio guidelines for submitting code & software for further information.

Data

Policy information about availability of data

All manuscripts must include a data availability statement. This statement should provide the following information, where applicable:

- Accession codes, unique identifiers, or web links for publicly available datasets
- A description of any restrictions on data availability
- For clinical datasets or third party data, please ensure that the statement adheres to our policy

All databases/links to websites used for this work are referred to in the Methods section or in the figure legends. The following publicly available datasets were used in this project: GRCh38 reference genome [https://www.ensembl.org/index.html]. High-throughput sequencing data generated in this study have been deposited in the GEO database under accession code GSE207453 [https://www.ncbi.nlm.nih.gov/geo/query/acc.cgi?acc=GSE207453]. Source data are provided with this paper. The data represented in the figures generated in this study are provided in the Supplementary Information/Source Data file. DDX5 RIP-seq data used in this study are available in the GEO database under accession code GSE175455 [https://www.ncbi.nlm.nih.gov/geo/query/acc.cgi?acc=GSE175455].

Human research participants

Policy information about studies involving human research participants and Sex and Gender in Research.

Reporting on sex and gender	N/A
Population characteristics	N/A
Recruitment	N/A
Ethics oversight	N/A

Note that full information on the approval of the study protocol must also be provided in the manuscript.

Field-specific reporting

Please select the one below that is the best fit for your research. If you are not sure, read the appropriate sections before making your selection.

☒ Life sciences ☐ Behavioural & social sciences ☐ Ecological, evolutionary & environmental sciences

For a reference copy of the document with all sections, see nature.com/documents/nr-reporting-summary-flat.pdf

Life sciences study design

All studies must disclose on these points even when the disclosure is negative.

Sample size	Sample size were determined based on common practice in the field, technical difficulty and throughput of the assay. The sample size (n) of each experiment is provided in the figure legends in the main manuscript and supplementary information files. We have generally adopted the good practice of running three independent biological replicates, when possible and especially for experiments comparing multiple groups we performed four biological replicates.
Data exclusions	No data points were excluded from analysis in any experiment depicted in this manuscript.
Replication	Every experiment was performed multiple times to ensure that the findings in this paper were reproducible. The number of replications for each experiment is indicated in figure legends.
Randomization	For cell culture-based experiments, cells were split from the same batch of cells and randomly divided for each treatment in each biological replicate.
Blinding	The investigators were not blinded to allocation during experiments and outcome assessment, as proper controls were already included during experiment design.

Reporting for specific materials, systems and methods

We require information from authors about some types of materials, experimental systems and methods used in many studies. Here, indicate whether each material, system or method listed is relevant to your study. If you are not sure if a list item applies to your research, read the appropriate section before selecting a response.

Materials & experimental systems

n/a	Involved in the study
☐	☒ Antibodies
☐	☒ Eukaryotic cell lines
☒	☐ Palaeontology and archaeology
☒	☐ Animals and other organisms
☒	☐ Clinical data
☒	☐ Dual use research of concern

Methods

n/a	Involved in the study
☒	☐ ChIP-seq
☐	☒ Flow cytometry
☒	☐ MRI-based neuroimaging

Antibodies

Antibodies used

Antibody name Supplier Catalog No. Dilution
Anti-m6A polyclonal antibody Abcam ab151230 1:100
Anti-METTL3 [EPR18810] monoclonal antibody Abcam ab195352 1:1000
Anti-METTL14 polyclonal antibody Atlas HPA038002 1:1000
Anti-ACTB-Peroxidase (AC-15) monoclonal antibody Sigma-Aldrich A3854 1:2500
Anti-YTHDC1 polyclonal antibody Abcam ab122340 1:500
Anti-DDX5 polyclonal antibody Cell Signalling 9877 1:1000
Anti-DDX5 polyclonal antibody Abcam ab10261 1:100
Anti-DROSHA polyclonal antibody Abcam ab12286 1:1000
Anti-SRSF3 polyclonal antibody Abcam ab125124 1:500
Anti-GAPDH (6C5) monoclonal antibody Santa Cruz Biotechnology sc-32233 1:1000
Anti-CCND1 (72-13G) monoclonal antibody Santa Cruz Biotechnology sc-450 1:500
Anti-c-MYC (9E10) monoclonal antibody Santa Cruz Biotechnology sc-40 1:500
Anti-ACTN (H-300) polyclonal antibody Santa Cruz Biotechnology sc-15335 1:1000
Anti-Rabbit IgG (H+L) Secondary Antibody, HRP Thermo Fisher Scientific 31460 1:10000
"Anti-Mouse IgG (H+L) Secondary Antibody, HRP " Thermo Fisher Scientific 32430 1:10000
Anti-Goat IgG (H+L) Secondary Antibody, HRP Thermo Fisher Scientific 31402 1:10000

Validation

All of the antibodies used in this study were commercial and suitable for specific purposes. The antibodies were validated based on the information from the manufacturer's instructions and were supported by multiple publications.

Eukaryotic cell lines

Policy information about cell lines and Sex and Gender in Research

Cell line source(s)

Wild-type human primary myoblasts (Telethon Biobank) were obtained from a skeletal muscle biopsy from a 2-year-old male child. Human ERMS RD cell line was derived from an embryonal rhabdomyosarcoma from a female patient and ARMS RH4 cells line was derived from an alveolar rhabdomyosarcoma from a female patient.

Authentication

No information is available about their authentication of wild-type human primary myoblasts. RD and RH4 cell lines were previously authenticated as described in Megiorni F, et al., Oncotarget. 2016.

Mycoplasma contamination

Cells were tested and resulted negative for Mycoplasma contamination.

Commonly misidentified lines
(See ICLAC register)

No commonly misidentified cell lines were used in this study.

Flow Cytometry

Plots

Confirm that:

☐ The axis labels state the marker and fluorochrome used (e.g. CD4-FITC).

☐ The axis scales are clearly visible. Include numbers along axes only for bottom left plot of group (a 'group' is an analysis of identical markers).

☐ All plots are contour plots with outliers or pseudocolor plots.

☒ A numerical value for number of cells or percentage (with statistics) is provided.

Methodology

Sample preparation

Cells were trypsinized and counted. An equal number of cells for each experimental condition was used for the analysis. Cells were washed once with PBS 1X (Sigma-Aldrich), fixed in 2 mL ice-cold 70% ethanol per 1x106 cells and incubated at 4°C overnight. Then cells were centrifuged for Smin at 300 x g at 4°C, washed once with PBS 1X (Sigma-Aldrich) and pelleted again. Cells were then resuspended in 300 µL PBS (Sigma-Aldrich) supplemented 100 µg/ml RNase A (Qiagen) and 50 µg/ml Propidium Iodide (Sigma-Aldrich), and then incubated in the dark for 30 minutes at room temperature.

Instrument	Samples were processed using a using a BD LSRFortessa (BD Biosciences, San Jose, CA, USA).
Software	The percentages of cells in different phases of the cell cycle were determined using the FlowJo V9.3.2 computer software (TreeStar, Ashland, OR, USA).
Cell population abundance	At least 10000 events for each sample were acquired.
Gating strategy	Gating was applied uniformly to all samples in order to exclude cells debries and doublets.

☐ Tick this box to confirm that a figure exemplifying the gating strategy is provided in the Supplementary Information.
